# Abundance, biomass and species richness of macrozoobenthos along an intertidal elevation gradient

**DOI:** 10.1002/ece3.10815

**Published:** 2023-12-14

**Authors:** Jana Dewenter, Joanne Yong, Peter J. Schupp, Kertu Lõhmus, Ingrid Kröncke, Stefanie Moorthi, Daniela Pieck, Lucie Kuczynski, Sven Rohde

**Affiliations:** ^1^ Institute for Chemistry and Biology of the Marine Environment (ICBM), Carl von Ossietzky Universität Oldenburg Oldenburg Germany; ^2^ Department for Marine Research Senckenberg am Meer Wilhelmshaven Germany; ^3^ Helmholtz Institute for Functional Marine Biodiversity (HIFMB), Carl von Ossietzky Universität Oldenburg Oldenburg Germany; ^4^ Institute of Biology and Environmental Sciences (IBU), Carl von Ossietzky Universität Oldenburg Oldenburg Germany

**Keywords:** East Frisian islands, elevational/depth diversity gradient, microphytobenthos, mud flats, tidal flats, Wadden Sea

## Abstract

Ecology aims to comprehend species distribution and its interaction with environmental factors, from global to local scales. While global environmental changes affect marine biodiversity, understanding the drivers at smaller scales remains crucial. Tidal flats can be found on most of the world's coastlines and are particularly vulnerable to anthropogenic disturbances. They are important transient ecosystems between terrestrial and marine ecosystems, and their biodiversity provides important ecosystem services. Owing to this unique, terrestrial–marine transition, strong environmental gradients of elevation, sediment composition and food availability prevail. Here, we investigated which regional and local environmental factors drive the spatio‐temporal dynamics of macrozoobenthos communities on back‐barrier tidal flats in the East Frisian Wadden Sea. On the regional level, we found that species composition changed significantly from west to east on the East Frisian islands and that total abundance and species richness decreased from west to east. On the local abiotic level, we found that macrozoobenthos biomass decreased with higher elevation towards the salt marsh and that the total abundance of organisms in the sediment significantly increased with increasing mud content, while biodiversity and biomass were not changing significantly. In contrast to expectations, increasing Chl *a* availability as a measure of primary productivity did not result in changes in abundance, biomass or biodiversity, but extremely high total organic carbon (TOC) content was associated with a decrease in biomass and biodiversity. In conclusion, we found regional and local relationships that are similar to those observed in previous studies on macrozoobenthos in the Wadden Sea. Macrozoobenthos biomass, abundance and biodiversity are interrelated in a complex way with the physical, abiotic and biotic processes in and above the sediment.

## Graphical abstract

This study investigates the spatio‐temporal dynamics of macrozoobenthos communities on back‐barrier tidal flats in the East Frisian Wadden Sea. They found significant changes in species composition and a decrease in abundance and species richness from west to east on the East Frisian Islands, while extremely high total organic carbon content was associated with a decrease in biomass and biodiversity, suggesting potential negative effects of nutrient inputs in tidal flat areas.

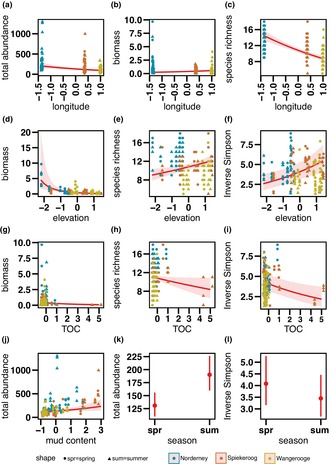

## 1 INTRODUCTION

One of the main aims in ecology is to understand the distribution of species and how they are influenced by abiotic and biotic environmental factors (Sutherland et al., 2013). Global environmental change has far‐reaching consequences for marine biodiversity and ecosystem functioning (Bulling et al., 2010; Dulvy et al., 2003; Worm et al., 2006), but predicting diversity and community composition at a global scale might not help to understand factors that are driving the diversity and community composition at smaller scales (Willis & Whittaker, 2002). Responses of marine macrozoobenthos communities to multiple drivers are far from being understood (Cheung et al., 2009; Hoegh‐Guldberg & Bruno, 2010; Ysebaert & Herman, 2002). At the regional scale, dispersal dynamics can play an important role in shaping the community composition and community properties (Palmer et al., 1996). On the local scale, environmental filtering, the prevention of establishment or persistence of an organism at a certain location can shape the community properties (Kraft et al., 2015). Furthermore, local food resource availability can also act as a filter by leading to the death of species (i.e., those unable to tolerate low food availability; Bauer et al., 2022). Additionally, biotic interactions such as competition and predation and positive trophic and non‐trophic interactions affect the communities (Bertness & Leonard, 2014; Ysebaert & Herman, 2002). Biotic interactions are changing with increasing temperatures, anthropogenic nutrient inputs, range expansion of or appearance of invasive species and exploitation by fishery (Beukema & Dekker, 2020). Deepening our understanding of the interplay of drivers affecting species richness, community composition, species traits and ecological functions is crucial to mitigate global change consequences on marine ecosystems by identifying structuring environmental factors across various spatial and temporal scales.

Coastal areas, including the Wadden Sea, are dynamic and unique ecosystems bridging the terrestrial and open sea ecosystems. Besides providing valuable economic (e.g., fish) and recreational ecosystem services (tourism), the large biomass of associated invertebrates supports the coastal food chain (de la Vega et al., 2018; Heip et al., 1995; Murray et al., 2019; Schückel et al., 2015; Sijtsma et al., 2019). The Wadden Sea comprises thousands of square meters of sand and mud flats located between barrier islands and the mainland coast (Essink et al., 1998; Flemming, 2012). Predatory fish and hundreds of thousands of migratory birds depend on macrozoobenthos as a food source and utilize this habitat as a nursery ground each year (Horn et al., 2020). Since 2009, the Wadden Sea has been declared a World Heritage Site due to its unique nature. Increasing habitat alteration (land reclamation and diking), exploitation (bottom trawling and shellfish dredging) and pollution (nutrients and chemicals) have changed the abundance and distribution of species in the Wadden Sea decreasing ecosystem functioning and the complexity of food webs (Eriksson et al., 2010; Lotze, 2005; Lotze et al., 2006). Furthermore, extreme weather events, expected to increase in frequency in the future, such as warm or unusually cold winters (ice‐winters), can lead to reduced recruitment and changes in the abundance of benthic species like bivalves and polychaetes (Bartels‐Hardege & Zeeck, 1990; Beukema & Dekker, 2005).

**FIGURE 1:**
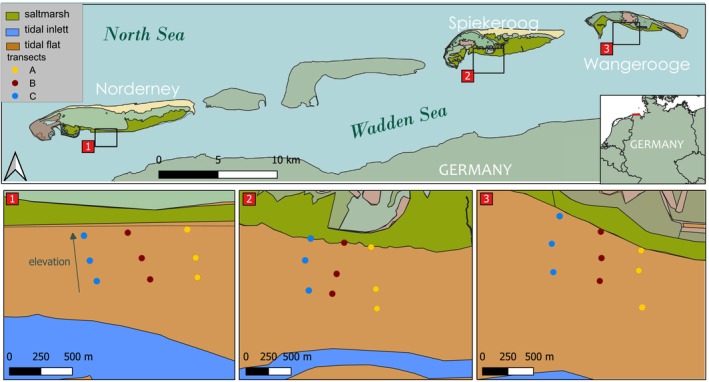
Map shows the three East Frisian islands, Norderney, Spiekeroog and Wangerooge with three transects (A = yellow, B = red and C = blue) at each island and three sample stations within each transect. At each sampling station, three samples were taken in spring and summer. The map was created with QGIS (version 3.24.1) and map data from OpenStreetMap.

Mostly east‐ward directed winds in the German Bight (Figure 1) induce the residual circulation to be cyclonic, going from west to east in the southern parts of the Wadden Sea (Otto et al., 1990; Staneva et al., 2009). During flood, the circulation is directed eastwards, changing to a westward circulation during ebb where water from the tidal flats is sucked out via the tidal channels (Meyerjürgens et al., 2019; Stanev et al., 2007). Hydrodynamic models and riverine nutrient inputs suggest a decreasing gradient of nutrient levels and possibly also larval supply from west to east along the Frisian islands, from Norderney over Spiekeroog to Wangerooge (Respondek et al., 2022; Stanev et al., 2003; van Beusekom et al., 2009, 2019), which could have an influence on the macrozoobenthos community composition at the islands.

Benthic macrozoobenthos is well adapted to the dynamics and harsh conditions of the tidal flats that are characterized by diurnal tides, and therefore constantly changing water levels, which leads to strong environmental gradients (Le Hir et al., 2000; Schückel et al., 2013; Sprung et al., 2001). Tidal flat organisms exhibit distinct distribution patterns within specific sections of the tidal flat, influenced by environmental gradients, primarily elevation, sediment composition and total organic carbon (Beukema & Dekker, 2009; Compton et al., 2013; Reise, 2002; Schückel et al., 2013; Ysebaert et al., 2003). Strong abiotic gradients induce a typical zonation of tidal flats with low species richness and high abundance of small organisms at the high‐tide line, close to the salt marsh (Beukema & Dekker, 2009). With decreasing elevation, species richness as well as biomass increase and then decline again towards exposed areas in the subtidal (Beukema & Dekker, 2009; Donadi et al., 2015; Schückel et al., 2013). Tidal flats can be classified into mud‐, mixed‐ and sand flats based on the content of mud or sand in the sediment (Flemming, 2000; Reineck & Siefert, 1980). Sediment composition can play a major role in structuring macrozoobenthos communities since many species are associated with a specific type of sediment, for example, for building tubes but also by reflecting the hydrodynamic regime and food availability (Armonies, 2021; Kröncke, 2006; Nehmer & Kröncke, 2003; Reise, 2002; Reiss & Kröncke, 2001; Schückel et al., 2013; Schückel & Kröncke, 2013). Overall, macrozoobenthos communities are predominantly influenced by two environmental factors: elevation and sediment.

Biotic conditions, such as primary production (Chl *a* biomass), can have influence on the macrobenthic communities by being a primary food source (Beukema & Cadée, 1997; Puls et al., 2012; Schückel et al., 2013). Next to seasonal variation, human activities, like increased nutrient run‐off from rivers, changes in hydrodynamics due to diking, loss of seagrass and bivalve fisheries, also influence primary production (Eriksson et al., 2010; Philippart et al., 2007). The Wadden Sea undergoes eutrophication and oligotrophication cycles (Burson et al., 2016; Lenhart et al., 2010; van Beusekom et al., 2019; van Raaphorst & de Jonge, 2004), affecting the total biomass and community composition of primary producers, which, in turn, cascade to higher trophic levels (Beukema & Dekker, 2022; Cloern, 2001; van Roomen et al., 2012). Nutrient availability is a central factor for the occurrence of microphytobenthos, consisting of unicellular eukaryotic algae and cyanobacteria (Hope et al., 2020; MacIntyre et al., 1996), and consequently, for the functioning of coastal soft‐sediment ecosystems. Microphytobenthos contributes to a large extent to the primary production of tidal flats and supports higher trophic levels, like macrozoobenthos, with essential fatty acids and energy by contributing large fractions to their nutrition (Christianen et al., 2017; Hope et al., 2020; MacIntyre et al., 1996). Microphytobenthos biomass generally increases with elevation and cohesiveness of the sediment, most likely due to the longer exposure time and therefore light availability, higher nutrient concentration and less resuspension of the sediment (Colijn & Dijkema, 1981; Underwood & Kromkamp, 1999; Yong et al., 2022). While opportunistic species do best in habitats with high organic enrichment, equilibrium species can better cope with lower concentrations (Linton & Taghon, 2000; Pearson & Rosenberg, 1978). Species performance in habitats with different levels of enrichment can depend on their physiological and behavioural adaptations (Linton & Taghon, 2000; Pearson & Rosenberg, 1978).

Despite the importance of understanding the distribution of macrozoobenthos on different spatial scales and under varying environmental conditions, data availability of the community composition of macrozoobenthos in combination with environmental and functional parameters (e.g., biomass) from the East Frisian islands is limited (Bergfeld, 1999; Dörjes et al., 1986; Hodapp et al., 2014; Kröncke, 1996; Nehmer & Kröncke, 2003; Reiss & Kröncke, 2001; Singer et al., 2023), and an evaluation of major environmental drivers across different islands is mainly missing.

The objective of this study is to quantify the abundance, biomass and diversity of benthic macrozoobenthos at both regional and local spatial levels, as well as in two seasons, in relation to abiotic and biotic environmental factors on tidal flats in the German Wadden Sea. Thus, we formulated the hypotheses as follows:

- H1: At the regional scale, the influence of dispersal limitation leads to fewer shared species between eastern and western communities, resulting in pronounced dissimilarity in their community composition.
- H2. Also, at the regional scale, nutrient‐induced species sorting, leads to a gradient in macrozoobenthos abundance, biomass and biodiversity, with higher levels observed in western areas compared with eastern areas.
- H3: Environmental filtering by local abiotic drivers influences macrozoobenthos characteristics, resulting in increasing abundance but decreasing biomass and diversity towards high‐tide line (Beukema & Dekker, 2009). Furthermore, with increasing mud content in the sediment, the total abundance (Armonies & Hellwig‐Armonies, 1987; van der Wal, Lambert, et al., 2017) and diversity of macrozoobenthos increase, and total biomass and species richness decrease (Bosco et al., 2022).
- H4: At the local scale, biotic filtering in terms of resource availability, displayed by microphytobenthos (Chl *a*) and TOC, causes an increase in abundance, biomass and diversity with increasing resource availability (Pearson & Rosenberg, 1978; Schückel et al., 2013). However, at extreme food availability, species richness and diversity decline, followed by a decrease in biomass due to an increase in abundant but small‐sized species (Pearson & Rosenberg, 1978). Additionally, recruitment of larvae in spring occurs at sites that provide suitable abiotic and biotic conditions, resulting in higher abundance and biomass in summer (Beukema, 1974). We have no particular expectations relative to diversity and season.

## 2 MATERIALS AND METHODS

### 2.1 Study area

The study took place in 2019 at the tidal flats of the East Frisian islands of Norderney (53.7078° N, 7.1431° E), Spiekeroog (53°45′728″ N, 7°43′367″ E) and Wangerooge (53.7890° N, 7.9020° E; Figure 1). The islands are part of the Wadden Sea National Park of Lower Saxony, located in the Southern North Sea of Germany. The tidal flats, which are on the southern side of the barrier islands, facing the mainland, are shaped by semidiurnal tides and relatively low current velocities (Hild et al., 1999).

### 2.2 Sample collection and analysis

#### 2.2.1 Macrozoobenthos community

To cover a broad range of environmental conditions (Table A2), we sampled a total of nine transects with 27 sampling stations, that is, three transects perpendicular to island coast at each island comprised of three sampling stations each. Each sampling station was marked with a DGPS to estimate the elevation of the study plot. The sampling stations covered a depth range from the high‐tide line to the middle‐tide line (Figure 1). To cover the spatial and temporal changes in the macrozoobenthos community, sampling took place once in spring and once in summer of 2019 (Table A1). At each of the sampling stations, we took three subsamples for macrozoobenthos with hand corers that had a diameter of 10 cm (0.0078 m^2^) and a height of 20 cm, which were driven into the sediment at low tide. The three cores were placed in separate plastic bags, transported to the shore and washed over a 500 μm sieve. Remaining organisms were then placed into separate plastic containers and fixated using 4% buffered‐formaldehyde solution for later identification. In the laboratory, samples were stained with rose bengal and identified to species level (or the lowest possible taxonomic unit) under a stereomicroscope (Leica MZ 95; Leitz, Wetzlar, Germany). For smaller organisms, an optical microscope (Leica DM LS 2) was used. After taxonomic identification, abundances of each species and wet biomass were determined. The World Register of Marine Species (WoRMS) taxon match tool was used to standardize the nomenclature (http://www.marinespecies.org/aphia.php?p=match). Not all organisms could be determined to species level; this was especially the case for oligochaetes, insect larvae and juvenile specimens. Therefore, species richness calculations were partly based on number of higher taxonomic units, resulting in a conservative estimation of diversity. Although only 15.2% of the specimens were identified to species level, we decided to still use the term species richness in data evaluation and discussion.

#### 2.2.2 Total organic carbon and sediment composition

To measure the total organic carbon (TOC) content, one surface sediment sample (<2 cm depth) per station was taken and frozen at −20°C until further analysis. The samples were then freeze‐dried in a Lyovac GT2 (STERIS GmbH, Huerth, Germany) and subsequently ground using a planetary ball mill (PM200; Retsch GmbH, Haan, Germany). Twenty‐five to seventy‐five milligrams of the material was weighed in silver cups and placed in a steaming 37%‐HCl desiccator for 24 h to remove carbonates. After that, one drop of 10%‐HCl was added to remove all carbonate leftovers. For evaporating the leftover HCl, the samples were placed in an oven at 50°C for 12 h. Subsequently, the silver cups were folded into small packages and analysed for total organic carbon in a CHNS‐Elemental‐Analyzer ‘vario EL cube’ using Loess as a standard (ELEMENTAR Analysensysteme GmbH Heraeus, Langenselbold, Germany).

To analyse the particle size distribution of the sediment at each station, a sediment sample of the upper 5 cm was taken with a hand corer (diameter 10 cm), placed in a plastic bag and frozen at −20°C until further analysis. Samples were thawed and mixed with a spatula and 20–35 g of sediment were dissolved in a 4%‐ sodium‐polyphosphate solution and sieved through a 2 mm sieve to remove large particles. To disperse the clay, the mixture was shaken overnight and subsequently measured with a laser diffraction spectrometer (HORIBA LA‐950). Sediment fraction of mud (<0.063 mm) was obtained in volume per cent.

#### 2.2.3 Inorganic nutrients and primary production

We used the dissolved inorganic nutrients and Chl *a* biomass informed by Yong et al. (2022), who took samples at the exact same locations and time as our macrozoobenthos samples. Dissolved inorganic nutrients were sampled using rhizon samplers (CSS 5 cm; 0.15 μm membrane pore size; 19.21.23F; Rhizosphere Research Products, Wageningen, Netherlands) that were inserted 2 cm in the sediment and connected to syringes. Samples were analysed for reactive dissolved phosphate (DIP) and dissolved inorganic nitrogen (ammonium, nitrite and nitrate combined) using a Scalar analytical auto‐analyzer (San++ System, Scalar Analytical, Breda, The Netherlands).

For Chl *a* determination, three sediment cores were taken at each sampling station from the upper 2 cm of the sediment with a cut‐off syringe (diameter 26 mm) and pooled together (total volume 31.85 cm^3^). In the field, samples were stored in the dark, cooled with ice packs and immediately frozen at −80°C after returning to the laboratory. The extraction and analysis of the pigment Chl *a* was performed after the method of Thrane et al. (2015) with slight modifications that can be found in Yong et al. (2022). In summary, Chl *a* was extracted from sediment subsamples (5 g) in 96% ethanol and the resulting supernatants were measured in triplicates using a Synergy MX plate reader (BioTek Instruments, Vermont, USA). Spectral scans were carried out between 300 and 800 nm with a resolution of 1 nm to measure Chl *a* concentrations, which is a proxy for total algal biomass. Results from the plate reader were analysed in R (R Core Team, 2021) with the R script provided by Thrane et al. (2015) that is based on a modified Gauss‐peak spectra (GPS) method.

### 2.3 Data analysis

Univariate and multivariate data were analysed using the program R (R Core Team, 2021; version 4.0.3 and RStudio Team, 2022).

**FIGURE 2:**
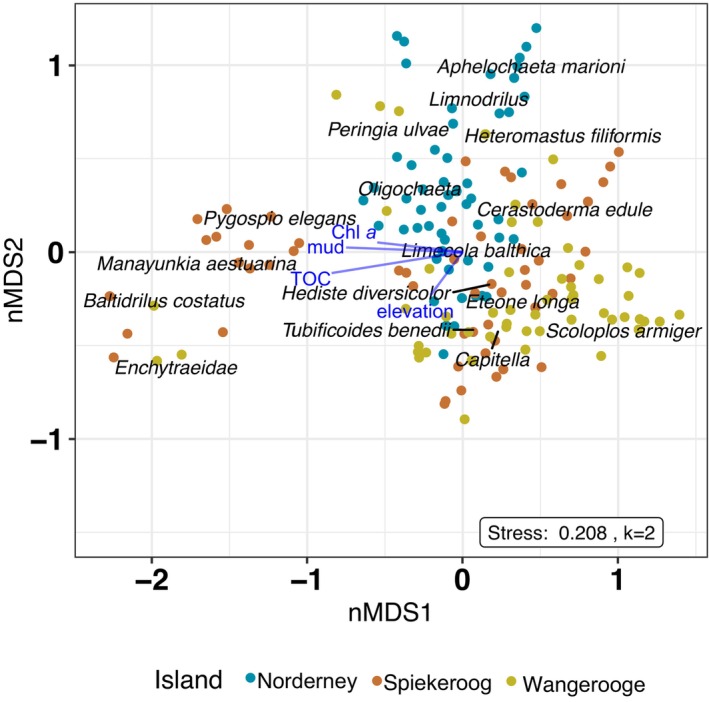
Non‐metric multidimensional scaling (NMDS) ordination diagram of the sampling stations (*n* = 162) at the three islands shown as colour coded (Nor = Norderney, Sp = Spiekeroog, Wang = Wangerooge). Effects of measured environmental variables on species assemblages of different East Frisian islands. Environmental drivers are indicated with vectors. The closer data points are to the centre the more similar they are in their species assemblage and the less they are influenced by the environmental parameters. Stress: 0.21, Dimension = 2.

Abundance data of macrozoobenthos in each subsample were standardized by total abundance of the subsample, because multivariate analysis can be sensitive to absolute abundances, and a Bray–Curtis dissimilarity matrix was calculated. To test the differences in community composition between islands (H1), we performed a PERMANOVA using the adonis2 function from the ‘vegan’ package with 9999 permutations (Oksanen et al., 2007). To visualize the similarity or dissimilarity of the macrozoobenthos communities, a non‐metric multidimensional scaling (NMDS) was performed. The resulting two‐dimensional ordination plot displays the samples sorted relative to their dissimilarity, with dissimilar samples further apart and similar samples closer together. For the NMDS, the function metaMDS from the ‘vegan’ package with 9999 permutations was used (Oksanen et al., 2007). In order to fit the environmental data onto the ordination, we used the envfit function from the ‘vegan’ package with 9999 permutations and rm.na set to true, to calculate the correlation of environmental variables with the ordination axes. Visualization was realized with ggplot2 (Wickham et al., 2016), and all significant environmental variables are shown as vectors in Figure 2, Table 1. We calculated species richness and the inverse Simpson diversity index using the function hill_taxa from the ‘hillR’ package (Chao et al., 2014; Li, 2018). Species richness (Hill number, *q* = 0), the inverse of Simpson's diversity index (Hill number, *q* = 2), the total number of individuals and the biomass were calculated per subsample. Data exploration was carried out following the protocol described by Zuur et al. (2010). Species richness and the inverse Simpson index were transformed logarithmically before analysis to ensure normality, while biomass was ln (*x* + 1) transformed. All fixed effects were scaled prior to analysis because fixed effects were on different scales.

**TABLE 1:** Results of PERMANOVA (adonis2), for macrozoobenthos relative abundances (Bray–Curtis dissimilarities, 9999 permutations) to investigate the effect of island on the macrozoobenthos community composition on the intertidal flats of the German Wadden Sea.

	df	SumOfSqs	*R* ^2^	*F*	Pr (>*F*)
Island	2	5.355	.129	11.775	1e‐04***
Residual	159	36.153	.871		
Total	161	41.507	.00		

*Note*: ***p < .001; **p < .01; *p < .05.

We fitted separate models for the response variables abundance, biomass, species richness and diversity using linear mixed effect models (LMMs) for normal distributed response variables (diversity and species richness) and generalized mixed effect models (GLMMs) for non‐normal distributed response variables (abundance and biomass) to test the hypothesis H2, H3 and H4 in combination using longitude (continuous), latitude (continuous), elevation (continuous), mud content (volume per cent), season (categorical), TOC (continuous) and Chl *a* (continuous) as explanatory variables. As link functions, we chose nbinom2, which is a negative binomial distribution with a quadratic parametrization, for the abundance (Hardin & Hilbe, 2018) and gaussian for species richness and inverse Simpson index. For biomass, which includes continuous but non‐negative values, we chose a tweedie distribution that was shown to be often appropriate for this kind of data (Foster & Bravington, 2013; Niku et al., 2017). For LMMs, the ‘lme4’ package (Bates et al., 2015) was used; for GLMMs, the ‘glmmTMB’ package was used (Brooks et al., 2017). Explanatory variables were checked for multicollinearity using variance inflation factors (VIF) obtained by the vif function from the ‘car’ package (Fox et al., 2012). VIFs indicated a high correlation of latitude and longitude. Therefore, we removed latitude as explanatory variable from the model. All remaining VIFs were lower than the threshold of 3 (Zuur et al., 2010). To make models converge, we used the ‘bobyqa’ optimizer which allows for a series of convergence checks. We increased the number of iterations to 200,000 using the ‘maxfun’ argument in the LMMs to help achieve convergence when the default value is not sufficient. In GLMMs, we used the optimizer ‘nlminb’ and increased iterations to 200,000. Marginal and conditional *R* ^2^ were calculated based on Johnson (2014), for assessing the goodness‐of‐fit.

To incorporate the dependency among observations from the same island and sampling station, we included island and sampling station as a random effect. However, the models for abundance, biomass and species richness as response variables resulted in singular fits, caused by the lack of spatial variables‐associated variance, and therefore, we simplified the random effect structure down to sampling station alone for these models (Barr et al., 2013). All models were checked for over‐ and underdispersion, zero‐inflation, homogeneity of variance and also the suitability of chosen error distributions using the *DHARMa* package (Hartig, 2022). To display the most important information of the models, the package ‘sjPlot’ was used (Lüdecke, 2022). To visualize the outcomes of the lmer and glmmTMB models, the predictions of the models were extracted using the ‘sjPlot’ package and combined with the raw data using the ‘ggplot2’ package (Wickham et al., 2016).

## 3 RESULTS

### 3.1 Spatial and temporal variability of macrozoobenthos

We collected and identified a total of 31,908 organisms comprising 45 different taxa overall (Table A3). Twenty‐five species of polychaetes, seven crustaceans, seven oligochaetes, five bivalves, two gastropods and one insect species were found during the study period. The species richness in subsamples varied between 5 and 18 (mean ± SD: 11.2 ± 2.84, *n* = 162).

### 3.2 Regional differences in the macrozoobenthos community composition (H1)

Macrozoobenthos communities differed significantly between islands (Figure 2), but only a small fraction of the variability was explained by the factor ‘island’ (PERMANOVA, *p* < .0001, *R* ^2^ = .129, Table 1). Norderney and Wangerooge communities clustered further apart from each other, while the community of Spiekeroog was in‐between (Figure 2), resembling the geographical distance of the islands. Some samples from Spiekeroog and Wangerooge clustered further apart from the rest of the samples. This part of the community was associated closely with the environmental factors mud, Chl *a* and TOC and the species *Manayunkia aestuarina*, *Enchytraeidae*, *Pygospio elegans* and *Baltidrilus costatus*. Other species such as *Scoloplos armiger* clustered on the opposite side of the mud vector and preferred sandy sites.

### 3.3 Effect of regional‐scale species sorting on abundance, biomass and diversity (H2)

**FIGURE 3:**
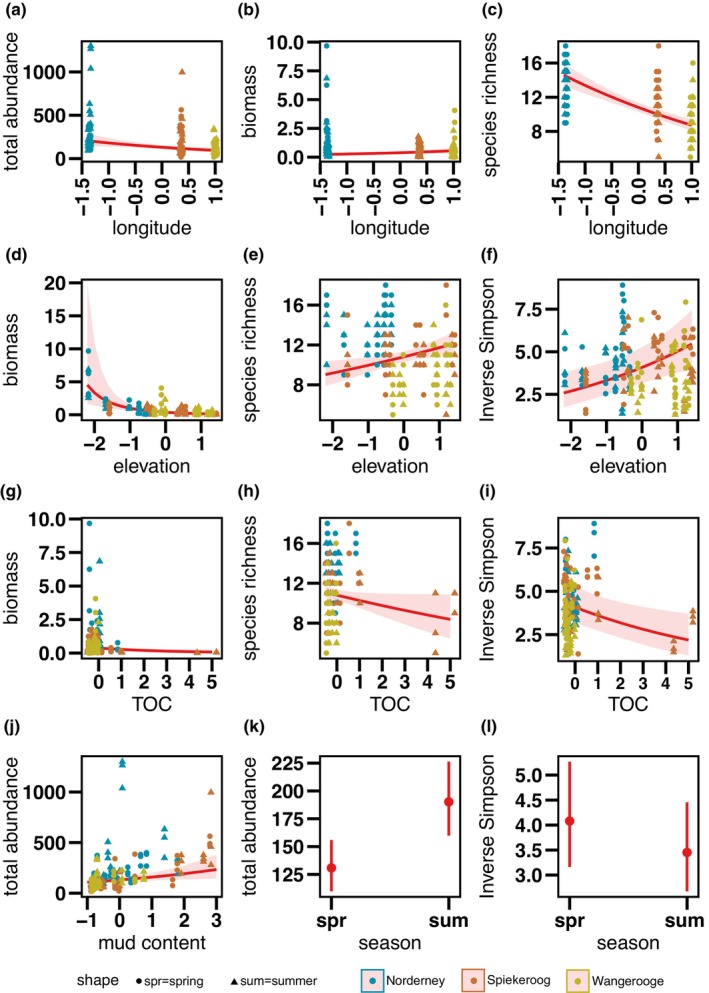
Effects of environmental drivers on macrozoobenthos abundance, biomass, species richness and diversity: (a) Relationship between longitude and total abundance, (b) longitude and biomass, (c) longitude and species richness, (d) elevation and biomass, (e) elevation and species richness, (f) elevation and inverse Simpson diversity, (g) TOC and biomass. (h) TOC and species richness, (i) TOC and inverse Simpson diversity, (j) mud content and total abundance, (k) season and total abundance, (l) season and inverse Simpson diversity. Shaded areas represent the 95% confidence intervals. Only the significant fixed effects of the model are shown here. The raw data are overlaid as data points, coloured in blue (Norderney), orange (Spiekeroog) and yellow (Wangerooge), round shapes are values from spring, triangles represent summer values. For details about the models see Table 2. Environmental variables.

Total abundance (Figure 3a, *p* = .002) and species richness (Figure 3c, *p* < .001) decreased significantly from west to east, while biomass showed a slight significant increase (Figure 3b, *p* = .014) in the opposite direction. Diversity showed no significant change from west to east (*p* = .121).

### 3.4 Relationship between abundance, biomass, species richness and diversity with local abiotic factors (H3)

Species richness and diversity increased significantly (Figure 3e,f, *p* = .005, *p* = .002, Table 2) with elevation towards the salt marsh, while biomass decreased significantly (Figure 3d, *p* < .001, Table 2). Total abundance showed no change with elevation (*p* = .986, Table 2). Sediment mud content had a significant positive effect on the total abundance (Figure 3j, *p* = .012, Table 2). Biomass, species richness and diversity depicted positive but non‐significant changes with mud content in the sediment (*p* = .43, *p* = .273, *p* = .16, Table 2).

**TABLE 2:** Results of models on biomass, species richness, abundance and inverse Simpson diversity over all islands and seasons in relation to environmental parameters (longitude, elevation, mud content, TOC, Chl *a* and season).

Predictors	Total abundance			Ln (biomass + 1)			Ln (species richness)			Ln (inverse Simpson)		
	Estimates	CI	*p*	Estimates	CI	*p*	Estimates	CI	*p*	Estimates	CI	*p*
(Intercept)	4.87	4.70 to 5.05	<.001***	−1.13	−1.34 to −0.92	<.001***	2.38	2.33 to 2.43	<.001***	1.41	1.15 to 1.67	<.001***
Longitude	−0.32	−0.53 to −0.12	.002**	0.31	0.06 to 0.55	.014*	−0.21	−0.27 to −0.16	<.001***	−0.20	−0.47 to 0.08	.157
Elevation	−0.00	−0.19 to 0.18	.986	−0.76	−1.00 to −0.51	<.001***	0.08	0.02 to 0.13	.007**	0.14	0.01 to 0.27	.030*
Mud	0.19	0.04 to 0.34	.012*	0.08	−0.12 to 0.27	.430	0.03	−0.02 to 0.08	.281	0.06	−0.04 to 0.16	.228
TOC	−0.01	−0.13 to 0.10	.822	−0.29	−0.51 to −0.08	.008**	−0.05	−0.10 to 0.00	.061	−0.11	−0.20 to −0.01	.023*
Chl *a*	0.05	−0.06 to 0.17	.351	0.17	−0.01 to 0.35	.068	−0.03	−0.07 to 0.01	.167	−0.08	−0.17 to 0.00	.064
Season [sum]	0.37	0.23 to 0.52	<.001***	−0.06	−0.29 to 0.17	.604	−0.00	−0.07 to 0.06	.905	−0.17	−0.28 to −0.06	.003**
Random effects												
*σ* ^2^	0.16			0.36			0.04			0.11		
*τ*_00_	0.15_station_id_			0.13_station_id_			0.00_station_id_			0.05_station_id_		
										0.04_island_		
ICC	0.48			0.27			0.10			0.46		
*N*	27_station_id_			27_station_id_			27_station_id_			3_island_		
										27_station_id_		
Observations	162			162			162			162		
Marginal *R* ^2^/Conditional *R* ^2^	.417/.697			.496/.632			.410/.469			.162/.546		
Family	nbinom2			Tweedie			Gaussian			Gaussian		

*Note*: The marginal *R* ^2^ only considers the variance of the fixed effects, so it stands for the proportion of variance explained by the model's fixed effects, while the conditional *R* ^2^ takes both the fixed and random effects into account. Similar to the *R* ^2^, the intra‐class correlation coefficients (ICCs) give information on how much of the proportion of variance is explained by a grouping (random) factor in multilevel/hierarchical data (Nakagawa et al., 2017). The random effect variance *σ* ^2^ stands for the mean random effect variance of the model. The random intercept variance, or between‐subject variance (*τ*_00_), indicates how much groups differ from each other. *N* stands for the number of random effect groups. The family nbinom2 is a negative binomial distribution with quadratic parameterization (Hardin & Hilbe, 2018). ***p < .001; **p < .01; *p < .05.

### 3.5 Effects of local biotic factors and season on the abundance, biomass, species richness and diversity of macrozoobenthos (H4)

Algal biomass (Chl *a*) had a non‐significant positive effect on total abundance and biomass (*p* = .351, *p* = .068, Table 2) and a non‐significant negative effect on species richness and diversity (*p* = .195, *p* = .161, Table 2). The amount of TOC in the sediment had a significant negative effect on the biomass (Figure 3g, *p* = .008, Table 2), species richness and diversity (Figure 3h,i, *p* = .046, *p* = .007, Table 2).

Seasonal changes were observed in total abundance, which increased significantly towards summer (Figure 3k, *p* < .001, Table 2), while at the same time, biomass showed a positive but non‐significant change towards summer (*p* = .068, Table 2). In contrast, the diversity declined significantly towards summer (Figure 3l, *p* = .003, Table 2) and species richness did not change significantly (*p* = .969, Table 2) with season.

Marginal *R* ^2^ values indicate how much of the variance is explained by the fixed effects while the conditional *R* ^2^ values explain the variance of both the fixed and the random effects. The marginal *R* ^2^ values in the models ranged between .187 and .496 and between .574 and .697 for conditional *R* ^2^ values (Table 2), indicating that the random effects also explained some of the variation in the models.

## 4 DISCUSSION

We investigated how community composition, abundance, biomass, species richness and diversity changed on the regional level from west to east and how local environmental factors (elevation, mud content, Chl *a* and TOC) and season affected these community properties. We found that (H1) species composition differed significantly between islands and (H2) that abundance and species richness decreased from west to east (H3). Biomass decreased with elevation, while diversity and species richness increased with elevation, while abundance increased with mud content (H4). Chl *a* showed no significant effects, but TOC affected biomass, species richness and diversity negatively. Total abundance increased from spring to summer, but inverse Simpson diversity decreased.

### 4.1 Regional‐scale dispersal limitation and nutrient‐induced species sorting: Implications for macrozoobenthos community composition and characteristics (H1)

Macrozoobenthos species composition might be shaped by dispersal limitations between the islands, resulting in significantly different species compositions at the islands and with western communities being less similar to more eastern communities (Figure 2, Table 1). Species composition of macrozoobenthos can be influenced by larval dispersal and larval settlement (Chust et al., 2016). Most macrozoobenthos species disperse via planktonic larvae but can also drift from one location to the other as juveniles or adults (Armonies, 1999). Therefore, the anticlockwise residual currents, the tides, the position of the tidal channels and the topography in the region (Stanev et al., 2003) could be one factor influencing the dispersal and recruitment of larvae (Bouma et al., 2001) and hence the community composition on the tidal flats of the islands. Another factor, known to affect macrozoobenthos community composition, is anthropogenic influences at the islands. After severe storm surges in the past, the East Frisian islands have been, to varying extents, protected with dikes, groynes and wooden barriers. While on Spiekeroog no coastal protection has been established, Norderney is protected with wooden barriers and Wangerooge is protected with groynes (NLWKN, 2010). The separation of salt marsh and mud flat by different flood protection measures might have contributed to the differences in community composition among the islands. Furthermore, local abiotic factors can also change with longitude, like the grain size and the mud content, and can have an influence on the community composition. Compton et al. (2013) found changes in the macrozoobenthos assemblages from west to east in the Dutch Wadden Sea that were explained mainly by mean grain size, microphytobenthos biomass and exposure time. Overall, functional traits, such as recruitment success, tolerance to abiotic conditions like the sediment characteristics (Anderson, 2008; Compton et al., 2009) or inundation times (Kraan et al., 2010) but also the local species pool and the food availability, can change the community composition (Beukema & Dekker, 2005; Schückel et al., 2013).

### 4.2 Regional changes of abundance, species richness and biomass (H2)

**FIGURE A1:**
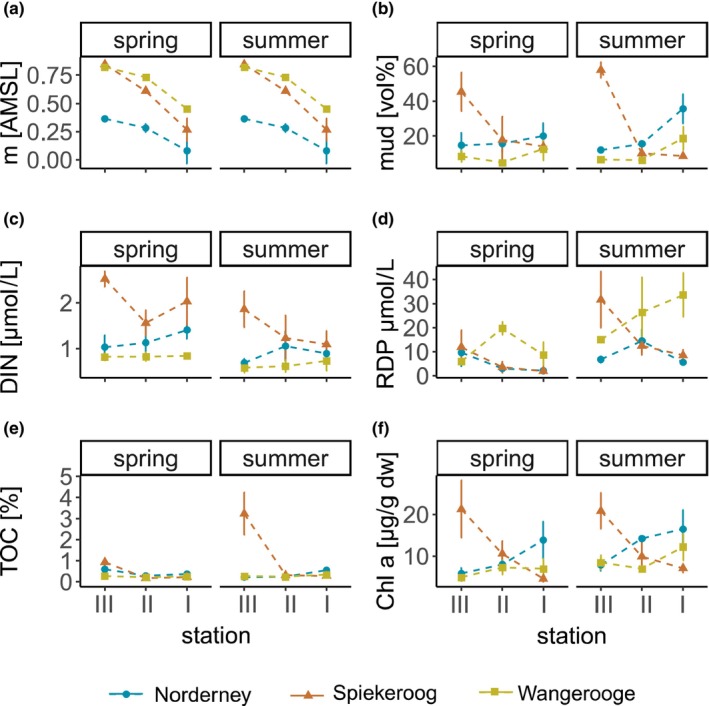
Variability of abiotic and biotic factors between seasons, stations and islands (a: elevation, b: mud percentage, c: dissolved inorganic nitrogen (DIN), d: reactive dissolved phosphate (RDP), e: total organic carbon (TOC), f: Chl *a*) in spring and summer at stations III, II and I Norderney (blue points), Spiekeroog (orange triangles) and Wangerooge (yellow squares).

We also found decreasing abundance and species richness from west to east. Abundance and species richness is like the community composition, influenced by dispersal (Mouquet & Loreau, 2003). Due to the prevailing hydrography, it is likely that the recruitment of different species is higher in the west compared with the east, thus increasing the abundance and diversity (Stanev et al., 2003). Even though the inverse Simpson index was not significantly affected by longitude, the direction of the trend was also negative (Table 2). Other than expected in hypothesis H2, biomass increased slightly towards the east. Compton et al. (2013) also observed increasing biomass from west to east in the Dutch Wadden Sea and attributed it to increasing microphytobenthos in the east, which is not the case in our study (Figure A1F). One possible explanation is the increase in suitable habitats for species dominating the biomass on tidal flats, such as the common cockle, *C. edule*, that prefers sandier sites (Figure 2; Kraan et al., 2010).

### 4.3 Impact of local abiotic drivers (mud content and elevation) on macrozoobenthos: Abundance, biomass and biodiversity patterns (H3)

The decrease in biomass with elevation partly supported H3. One of the reasons, for the biomass decline might be due to the higher abundance of larger bivalves at mean‐tide level (Beukema & Dekker, 2009), often contributing in large proportions to the biomass of tidal flats (Daggers et al., 2020; Essink et al., 1998). Often, filter feeders like *C. edule* profit from longer inundation times at lower elevation levels having more time to filter phytoplankton, as well as surface (deposit) feeders, which are mostly restricted to times of inundation to feed (Beukema & Dekker, 2009; Van Colen et al., 2010). A relatively small number of species were responsible for a similar pattern in a study from Hodapp et al. (2014) including species such as *C. edule*, *H. diversicolor* and *H. filiformis*. The dominant species at low elevations are characterized by relatively high body mass, which might explain the increase in biomass without an increase in total abundance or species richness towards lower elevations.

We observed significant positive changes in species richness and diversity with increasing elevation. This pattern contrasted with the traditional reported zonation of tidal flats where diversity is low near the salt marsh and increases towards low tide levels (Beukema & Dekker, 2009). Species at the border of the salt marsh tend to be characterized by small body size. Many studies investigating the zonation of macrozoobenthos, used a mesh size of 1 mm (e.g., Beukema & Dekker, 2009), while a mesh size of 0.5 mm was used in the current study. The study of Bachelet (1990) compared the efficacy of different mesh sizes and found that only 15% of polychaetes were captured in studies using 1 mm mesh size. Sieves with 0.5 mm mesh, like in this study, resulted in 53% of capture of polychaetes (Bachelet, 1990; Reise et al., 1994). This could result in different patterns of species richness and diversity in intertidal flats. The transition between tidal flat and salt marsh, is also a transition zone between two ecological communities called ecotone (Bearup & Blasius, 2017). Ecotones are often areas were species of two different communities overlap, which can lead to higher biodiversity compared with the adjacent habitats (Kark & van Rensburg, 2006), which could also be the case here.

The tidal flats on Wangerooge were characterized as sandy (mud content <10%), while the other two islands have mixed sediment types (mud content 10–50%; Flemming & Davis Jr., 1994). Even though mud content did not explain total biomass, species richness and diversity as we expected in H4, it had a significant positive effect on the total abundance in our study (Figure 3j, Table 2). Therefore, H4 can be partly accepted, as abundance increased with increasing mud content. This indicates a shift to a higher abundance of smaller species, which was also demonstrated by the dominance of oligochaetes and small polychaetes like *Baltidrilus costatus* and *Manayunkia aestuarina* at sites with high mud content. This pattern is particularly strong for Wangerooge, where a high sand content and the lack of the typical zonation with higher mud content at the shore caused by higher hydrodynamic stress resulted in lower macrozoobenthos abundance and species richness (Friedrichs, 2012; Schückel et al., 2013; van der Wal, Ysebaert, & Herman, 2017). Overall, human modifications of the coast caused current velocities to increase, which has led to a depletion of fine‐grained sediment (Dellwig et al., 2000). Increased macrozoobenthos abundances with higher mud content have also been reported by other studies (Armonies & Hellwig‐Armonies, 1987; van der Wal, Lambert, et al., 2017). Armonies and Hellwig‐Armonies (1987) found that abundance increased significantly towards the shore when sediments were muddy in contrast to other sediment types, where abundance peaks at mean high tide or was not increasing. However, it is important to note that this pattern is not universally valid. Beukema (1976) found the highest species richness at intermediate mud content and tidal level in the Dutch Wadden Sea.

Bosco et al. (2022) investigated how different diversity metrics and biomass changed along a sediment texture gradient and found changes for all parameters with mud content and sediment grain size. There are some methodological differences between the study of Bosco et al. (2022) and our study: Simpson diversity was calculated based on biomass and not abundance, GAMs were used to investigate the relationship, and in our study, the mud content had a broader range. Several studies found that macrozoobenthos species have specific preferences for certain sediment types (Anderson, 2008), resulting in a unimodal response of species richness to sediment grain size, which can be explained by an overlap of species that prefer muddy or sandy habitats at intermediate level grain sizes (Bosco et al., 2022). It is important to keep in mind that next to environmental filtering there are also other abiotic processes that can influence the macrozoobenthos community structure, for example, the spatial heterogeneity of abiotic factors (Kraft et al., 2015).

### 4.4 Impact of local biotic filters (Chl *a*, TOC) and seasonality on macrozoobenthos: Abundance, biomass and biodiversity patterns (H4)

We expected that sampling stations with high but not extreme resource availability in terms of Chl *a* and TOC would support a higher abundance, biomass and diversity of macrozoobenthos, as microphytobenthos and organic carbon are the major food sources of macrozoobenthos (Christianen et al., 2017; Daggers et al., 2020; Riekenberg et al., 2022). We cannot confirm part of H4, as we could not observe any relationship of the community characteristics with Chl *a* (Table 2). Following the SAB model of Pearson and Rosenberg (1978), we expected that extreme values of TOC would lead to a decline in abundance, biomass, species richness and diversity, which we can confirm for all parameters except for abundance (Figure 3g–i, Table 2). Abundance is expected to peak shortly before extreme enrichment, as more opportunistic, small‐sized species become abundant (Pearson & Rosenberg, 1978; Van Colen et al., 2012), and this might be the reason we did not observe any relationship between abundance and resource availability.

A study from Drent (2010) also could not link summer Chl *a* levels from the water column to biomass, abundance and diversity of macrozoobenthos. Unlike the Drent study which used phytoplankton as resource proxy, we used microphytobenthos for resource availability. Despite microphytobenthos being the most important food source for many macrozoobenthos species (except for filter feeders and predators; Christianen et al., 2017; Daggers et al., 2020; Lange et al., 2018), we found no significant effects. Furthermore, food resources are not always the limiting factor at all elevations. At high elevations where the biomass of macrozoobenthos is low, the corresponding food demand is also low. Consequently, no food shortage is present, which makes other environmental factors more important (Beukema et al., 2002). It is also plausible that few superior competitive macrozoobenthos species dominate at the most productive environments, resulting in the observed negative relationship between macrozoobenthos species richness and productivity (Kondoh, 2001).

Additionally, factors such as the actual consumption rates of macrozoobenthos and the productivity of macrozoobenthos and microphytobenthos would be of interest, since top‐down control by grazing from meiofauna and macrozoobenthos can influence the Chl *a* biomass at a site (Daggers et al., 2020). Information from these factors would reveal not only the existent resource availability, but also the total resource availability without consumption by other trophic levels. Top‐down control from macrozoobenthos on microphytobenthos can change the spatial patterns of microphytobenthos in intertidal mud flats as shown in field experiments (Weerman et al., 2011). The decrease in microphytobenthos coincided with an increase in the abundance of macrozoobenthos (Weerman et al., 2011).

We can partly confirm H4 as we found increasing abundances from spring to summer (Figure 3k, Table 2). Spawning and larvae settlement in spring results in higher abundances in summer when young specimen had time to grow to a size that was retained in sieves with a mesh size of 0.5 mm (Beukema & de Vlas, 1989). We could not detect any changes in biomass, probably because freshly settled individuals are still small, contributing only minor amounts to the total biomass (Beukema, 1974). Other factors, such as individual weight gains (Beukema, 1974), migration or the vulnerability of species sensitive to high summer temperatures (Beukema & Dekker, 2020), could act as balancing forces in influencing changes in biomass. We have to partly reject H4, as we did not predict that diversity decreased during summer. The drop in diversity can be a consequence of unusually high summer temperatures in 2019 (Meier et al., 2020; Zielinski et al., 2019), which might have caused migration or die‐off of some species sensitive to high temperatures (Beukema et al., 2009).

Bearing in mind that macrozoobenthos species themselves have important ecosystem functions like bioturbation, destabilization or stabilization or reef formation that can influence the sediment characteristics (Eriksson et al., 2010; Meysman et al., 2006; Reise, 2002), physical, biochemical properties and hydrodynamics of their surroundings. Additionally, it is still unclear whether biotic resource filters, such as microphytobenthos Chl *a* biomass and TOC, which are consumed by macrozoobenthos, are good proxies for food availability (Hagerthey et al., 2002; Pratt et al., 2015; Weerman et al., 2011). Thus, it is difficult to disentangle the cause‐and‐effect dynamics of environmental filters and resource filters on macrozoobenthos communities.

## 5 CONCLUSION

In conclusion, our research revealed new insights on how benthic macroinvertebrate abundance, biomass, species richness and diversity change with regional as well as local abiotic and biotic environmental filters in an important boundary zone between land and sea. In view of climate change and rising sea levels, environmental abiotic and biotic conditions are expected to change in back‐barrier tidal flats in terms of sediment composition, resource availability, elevation and extreme weather events. Interestingly, environmental factors shaped not only diversity patterns and dynamics of intertidal benthic macrozoobenthos on back‐barrier tidal flats in the Wadden Sea but also community composition. As abiotic factors often covary, but with contrasting impacts on the macrozoobenthos community, it is challenging to entangle which abiotic factors are influencing the macrozoobenthos community in which way, especially when relying on correlative data. The regional and local relationships of biomass, abundance and diversity we observed are similar to those found in previous studies on macrozoobenthos in the Wadden Sea.

Despite its protection status, the Wadden Sea exists close to human pressures. Negative impacts do not need to have their source inside the protected area, which is for example the case for nutrient inputs from rivers (Hill et al., 2021). Management and protection of tidal flats worldwide have to be improved and monitored to protect the biodiversity and unique species assemblage that can be found in these coastal ecosystems.

## AUTHOR CONTRIBUTIONS

**Jana Dewenter:** Conceptualization (equal); data curation (lead); formal analysis (lead); investigation (lead); project administration (equal); visualization (lead); writing – original draft (lead). **Joanne Yong:** Conceptualization (supporting); data curation (equal); investigation (supporting); project administration (supporting); resources (supporting); writing – review and editing (supporting). **Peter J. Schupp:** Conceptualization (equal); funding acquisition (supporting); project administration (supporting); resources (equal); writing – review and editing (equal). **Stefanie Moorthi:** Conceptualization (supporting); funding acquisition (supporting); methodology (supporting); resources (supporting); supervision (supporting); writing – review and editing (supporting). **Kertu Lõhmus:** Project administration (supporting); resources (supporting); writing – review and editing (supporting). **Ingrid Kröncke:** Conceptualization (equal); funding acquisition (equal); project administration (equal); resources (equal); writing – review and editing (equal). **Daniela Pieck:** Project administration (supporting); resources (supporting); writing – review and editing (supporting). **Lucie Kuczynski:** Conceptualization (supporting); formal analysis (supporting); supervision (supporting); writing – review and editing (supporting). **Sven Rohde:** Conceptualization (equal); funding acquisition (lead); project administration (equal); resources (equal); supervision (equal); writing – review and editing (lead).

## CONFLICT OF INTEREST STATEMENT

The authors declare that they have no known competing financial interests or personal relationships that could have appeared to influence the work reported in this paper.

## Data Availability

The data that support the findings of this study are openly available in DRYAD at Dewenter et al. (2023). Data at the moment accessible under: https://datadryad.org/stash/share/0SWEkX‐fvDE9R4Xi‐x6KjstKFXb3Pv9MdXenOVhCoIw.

## APPENDIX A

**TABLE A1:** Sampling dates at the different islands in spring and summer 2019.

Island	Season	Sampling dates (DD/MM/YY)
Norderney	Spring	29/04/2019–30/04/2019
	Summer	01/07/2019–03/07/2019
Spiekeroog	Spring	15/04/2019–18/04/2019
	Summer	15/07/2019–18/07/2019
Wangerooge	Spring	16/05/2019–17/05/2019
	Summer	29/07/2019–30/07/2019

**TABLE A2:** Summary of environmental variables at the different islands and sampling stations.

	Norderney			Spiekeroog			Wangerooge		
	III (*N* = 6)	II (*N* = 6)	I (*N* = 6)	III (*N* = 6)	II (*N* = 6)	I (*N* = 6)	III (*N* = 6)	II (*N* = 6)	I (*N* = 6)
Mean grain size									
Min	131.41	172.62	128.47	75.42	127.91	225.18	211.72	232.35	140.44
Max	221.10	223.08	214.65	156.98	254.61	263.67	238.61	252.33	259.24
Mean (SD)	192.65 ± 31.83	202.95 ± 16.73	169.60 ± 34.41	106.07 ± 30.87	208.63 ± 45.97	241.73 ± 13.61	224.35 ± 9.93	241.15 ± 7.24	203.34 ± 48.39
Mud									
Min	4.93	6.43	6.55	25.64	3.44	7.62	3.67	3.91	5.68
Max	28.57	23.67	47.37	64.20	44.76	17.55	14.95	6.47	30.13
Mean (SD)	13.28 ± 8.07	15.53 ± 5.67	27.90 ± 14.96	51.69 ± 14.71	13.84 ± 16.09	11.13 ± 4.30	7.42 ± 3.91	5.36 ± 0.95	15.57 ± 10.89
Chl *a*									
Min	4.45	5.05	7.85	14.03	5.65	3.10	3.96	4.23	4.38
Max	10.49	15.09	25.03	34.88	16.95	9.03	11.91	10.21	16.41
Mean (SD)	6.84 ± 2.37	11.11 ± 3.87	15.16 ± 7.11	21.04 ± 8.81	10.22 ± 5.14	5.82 ± 1.94	6.68 ± 2.89	7.12 ± 2.05	9.59 ± 5.21
TOC									
Min	0.14	0.20	0.19	0.61	0.12	0.20	0.16	0.17	0.14
Max	1.16	0.40	0.59	4.55	0.47	0.36	0.48	0.37	0.49
Mean (SD)	0.41 ± 0.38	0.27 ± 0.08	0.46 ± 0.14	2.08 ± 1.68	0.24 ± 0.13	0.25 ± 0.06	0.28 ± 0.12	0.23 ± 0.08	0.30 ± 0.13
Temperature									
Min	10.9	10.9	11.3	NA	NA	NA	12.1	12	12
Max	22.4	22.2	22.2	NA	NA	NA	26.3	26.4	27
Mean (SD)	15.68 ± 4.95	15.78 ± 4.61	16.20 ± 4.52	4; 17.10 ± 1.18	4; 17.38 ± 1.06	17.77 ± 1.87	18.87 ± 6.89	18.78 ± 7.09	18.58 ± 7.19
DIN									
Min	0.52	0.57	0.71	1.22	0.6	0.75	0.4	0.36	0.41
Max	1.54	1.75	1.7	2.77	2.2	2.66	0.98	0.96	1.14
Mean (SD)	0.86 ± 0.35	1.09 ± 0.52	1.15 ± 0.37	2.19 ± 0.58	1.40 ± 0.65	1.56 ± 0.82	0.70 ± 0.20	0.72 ± 0.21	0.79 ± 0.24
RDP									
Min	1.39	1.31	1.6	3.67	0.77	1.05	4.63	9.98	2.19
Max	20.55	23.74	7.3	55.04	17.48	12.4	17.07	55.17	50.82
Mean (SD)	8.20 ± 6.58	8.74 ± 8.16	3.83 ± 2.23	21.81 ± 18.49	7.99 ± 6.83	5.21 ± 4.40	10.46 ± 5.13	23.08 ± 16.50	21.16 ± 17.90
Elevation									
Min	0.34	0.22	−0.09	0.81	0.58	0.07	0.81	0.71	0.41
Max	0.40	0.34	0.29	0.87	0.64	0.38	0.84	0.74	0.49
Mean (SD)	0.36 ± 0.03	0.28 ± 0.06	0.08 ± 0.17	0.85 ± 0.03	0.61 ± 0.03	0.27 ± 0.16	0.82 ± 0.02	0.73 ± 0.01	0.45 ± 0.04

**TABLE A3:** Mean abundance and standard deviation and mean biomass and standard deviation per island and species per core (0.0078 m^2^).

Species	Island	Mean abundance ± SD	Mean biomass ± SD	*n*
*Alitta succinea*	Norderney	2.111 ± 1.364	0.151 ± 0.117	9
*Alitta succinea*	Spiekeroog	1 ± NA	0.064 ± NA	1
*Alitta succinea*	Wangerooge	2 ± NA	0.32 ± NA	1
*Aphelochaeta marioni*	Norderney	119.222 ± 145.304	0.052 ± 0.06	18
*Aphelochaeta marioni*	Spiekeroog	19.182 ± 30.616	NA ± NA	11
*Aphelochaeta marioni*	Wangerooge	6.4 ± 7.403	0.001 ± 0.001	5
*Aphelochaeta* sp.	Spiekeroog	6 ± NA	0.005 ± NA	1
*Aphelochaeta* sp.	Wangerooge	2 ± NA	0 ± NA	1
*Arenicola marina*	Norderney	2.857 ± 2.911	0.427 ± 0.625	7
*Arenicola marina*	Spiekeroog	1.286 ± 0.756	1.455 ± 2.214	7
*Arenicola marina*	Wangerooge	1.333 ± 0.577	1.359 ± 2.164	3
*Baltidrilus costatus*	Norderney	34.5 ± 17.678	0.015 ± 0.002	2
*Baltidrilus costatus*	Spiekeroog	207.571 ± 150.116	0.147 ± 0.102	7
*Baltidrilus costatus*	Wangerooge	21.667 ± 24.685	0.014 ± 0.019	3
*Bathyporeia elegans*	Wangerooge	2 ± NA	0.008 ± NA	1
*Bathyporeia sarsi*	Norderney	1 ± NA	0.006 ± NA	1
*Bathyporeia sarsi*	Spiekeroog	2.286 ± 1.113	0.006 ± 0.007	7
*Bathyporeia sarsi*	Wangerooge	2.25 ± 1.893	0.004 ± 0.003	4
*Bathyporeia* sp.	Wangerooge	1 ± NA	0.001 ± NA	1
*Bathyporeia* sp. juv	Spiekeroog	1.667 ± 0.577	0 ± 0	3
*Bathyporeia* sp. juv	Wangerooge	1 ± 0	0 ± 0	3
*Bivalvia* sp. juv	Norderney	2 ± 1.414	0.006 ± 0.015	8
*Bivalvia* sp. juv	Spiekeroog	7 ± 5.657	0.009 ± 0.002	2
*Bivalvia* sp. juv	Wangerooge	6.25 ± 6.85	0.004 ± 0.008	4
*Capitella capitata*	Norderney	11 ± 13.077	0.024 ± 0.025	3
*Capitella capitata*	Spiekeroog	12 ± 9.925	0.041 ± 0.038	5
*Capitella capitata*	Wangerooge	9.143 ± 5.521	0.064 ± 0.104	7
*Capitella* sp.	Norderney	20.125 ± 20.266	0.014 ± 0.014	8
*Capitella* sp.	Spiekeroog	5.571 ± 4.036	0.007 ± 0.01	7
*Capitella* sp.	Wangerooge	29.889 ± 30.824	0.029 ± 0.025	9
*Carcinus maenas*	Norderney	2 ± 1.225	0.018 ± 0.012	5
*Carcinus maenas*	Spiekeroog	3.667 ± 3.204	0.061 ± 0.058	6
*Carcinus maenas*	Wangerooge	1 ± 0	0.022 ± 0.015	5
*Cerastoderma edule*	Norderney	22.647 ± 16.14	NA ± NA	17
*Cerastoderma edule*	Spiekeroog	18.154 ± 15.768	19.839 ± 13.797	13
*Cerastoderma edule*	Wangerooge	14.923 ± 29.125	19.482 ± 27.367	13
*Cerastoderma edule juv*	Norderney	4.25 ± 4.743	0.018 ± 0.025	8
*Cerastoderma edule juv*	Spiekeroog	10.6 ± 7.668	0.09 ± 0.117	5
*Cerastoderma edule juv*	Wangerooge	4.833 ± 4.215	0.124 ± 0.138	6
*Cirrophorus* sp.	Norderney	12 ± NA	0.018 ± NA	1
*Cirrophorus* sp.	Wangerooge	1 ± NA	0.002 ± NA	1
*Coleoptera* sp.	Wangerooge	1 ± NA	0 ± NA	1
*Corophium arenarium*	Norderney	9.222 ± 7.429	0.011 ± 0.007	9
*Corophium arenarium*	Spiekeroog	2 ± 1.118	0.009 ± 0.007	9
*Corophium arenarium*	Wangerooge	6.214 ± 8.368	0.01 ± 0.01	14
*Corophium* sp.	Wangerooge	2 ± NA	0 ± NA	1
*Corophium* sp. juv	Wangerooge	1.8 ± 1.304	0 ± 0	5
*Crangon crangon*	Norderney	2 ± 2.236	0.021 ± 0.02	5
*Crangon crangon*	Spiekeroog	1.4 ± 0.894	0.011 ± 0.007	5
*Crangon crangon*	Wangerooge	1 ± 0	0.007 ± 0.009	2
*Dolichopodidae larvae*	Spiekeroog	14.333 ± 10.033	0.036 ± 0.026	6
*Dolichopodidae larvae*	Wangerooge	3.667 ± 2.887	0.009 ± 0.002	3
*Enchytraeidae* sp.	Spiekeroog	207 ± 404.302	0.027 ± 0.043	9
*Enchytraeidae* sp.	Wangerooge	95 ± 131.522	0.036 ± 0.05	2
*Eteone longa*	Norderney	22.778 ± 19.717	0.026 ± 0.029	18
*Eteone longa*	Spiekeroog	11.333 ± 13.292	0.015 ± 0.016	15
*Eteone longa*	Wangerooge	21 ± 15.58	0.01 ± 0.007	17
*Eumida sanguinea Reste*	Norderney	1 ± NA	0 ± NA	1
*Eumida* sp.	Norderney	1 ± NA	0 ± NA	1
*Eunereis longissima*	Norderney	5.6 ± 5.358	0.093 ± 0.088	10
*Eunereis longissima*	Spiekeroog	3 ± 2.749	0.088 ± 0.129	10
*Eunereis longissima*	Wangerooge	4 ± 2.966	0.241 ± 0.292	6
*Eunereis* sp.	Wangerooge	1 ± NA	0.114 ± NA	1
*Grania* sp.	Spiekeroog	1.667 ± 1.155	0.002 ± 0.003	3
*Hediste diversicolor*	Norderney	6.765 ± 3.833	0.774 ± 0.697	17
*Hediste diversicolor*	Spiekeroog	5.667 ± 4.923	0.969 ± 1	15
*Hediste diversicolor*	Wangerooge	4.667 ± 5.315	0.531 ± 0.413	9
*Hediste diversicolor juv*	Norderney	1 ± NA	0.001 ± NA	1
*Hediste diversicolor juv*	Spiekeroog	1 ± NA	0.007 ± NA	1
*Hediste diversicolor juv*	Wangerooge	2.75 ± 1.5	0.002 ± 0.002	4
*Heteromastus filiformis*	Norderney	55.111 ± 69.908	0.903 ± 1.003	18
*Heteromastus filiformis*	Spiekeroog	5.8 ± 3.425	0.158 ± 0.166	10
*Heteromastus filiformis*	Wangerooge	5.8 ± 6.563	0.074 ± 0.083	10
*Lanice conchilega*	Norderney	1.5 ± 0.577	0.156 ± 0.178	4
*Limecola balthica*	Norderney	5.417 ± 4.852	0.54 ± 0.326	12
*Limecola balthica*	Spiekeroog	2.583 ± 1.832	0.447 ± 0.488	12
*Limecola balthica*	Wangerooge	3 ± 4.147	0.406 ± 0.374	11
*Limecola balthica juv*	Norderney	19.733 ± 14.959	0.033 ± 0.039	15
*Limecola balthica juv*	Spiekeroog	17.294 ± 16.777	0.035 ± 0.047	17
*Limecola balthica juv*	Wangerooge	23.312 ± 30.888	0.057 ± 0.134	16
*Limnodrilus* sp.	Norderney	75.4 ± 136.746	0.026 ± 0.054	15
*Limnodrilus* sp.	Spiekeroog	30.5 ± 47.91	0.018 ± 0.031	10
*Limnodrilus* sp.	Wangerooge	56.25 ± 68.588	0.021 ± 0.025	4
*Magelona johnstoni*	Norderney	1 ± NA	0.003 ± NA	1
*Manayunkia aestuarina*	Spiekeroog	111.857 ± 139.837	0.014 ± 0.02	7
*Monopylephorus irroratus*	Spiekeroog	2 ± NA	0.006 ± NA	1
*Mya arenaria*	Norderney	1 ± NA	0.314 ± NA	1
*Nephtys caeca*	Norderney	1 ± NA	0.247 ± NA	1
*Nephtys hombergii*	Spiekeroog	1 ± NA	0.695 ± NA	1
*Nephtys* sp. juv	Norderney	1 ± NA	0.002 ± NA	1
*Nereididae larvae*	Norderney	16.571 ± 15.458	0.004 ± 0.004	7
*Nereididae larvae*	Spiekeroog	12.75 ± 22.838	0.001 ± 0	4
*Nereididae larvae*	Wangerooge	7.571 ± 3.552	0.003 ± 0.003	7
*Oligochaeta* sp.	Norderney	32 ± 60.017	0.008 ± 0.014	4
*Oligochaeta* sp.	Spiekeroog	23.4 ± 26.111	0.016 ± 0.025	5
*Oligochaeta* sp.	Wangerooge	12 ± 9.165	0.002 ± 0.002	3
*Peringia ulvae*	Norderney	328.706 ± 715.989	0.446 ± 0.488	17
*Peringia ulvae*	Spiekeroog	33.357 ± 36.038	0.338 ± 0.514	14
*Peringia ulvae*	Wangerooge	42.267 ± 61.401	0.305 ± 0.397	15
*Pholoe baltica*	Wangerooge	1 ± NA	0 ± NA	1
*Phyllodoce mucosa*	Norderney	2.667 ± 2.658	0.007 ± 0.009	6
*Phyllodoce mucosa*	Spiekeroog	1.333 ± 0.577	0.019 ± 0.025	3
*Phyllodoce mucosa*	Wangerooge	1 ± 0	0.007 ± 0.004	3
*Polydora cornuta*	Spiekeroog	4 ± NA	0.046 ± NA	1
*Pygospio elegans*	Norderney	60.294 ± 98.939	0.06 ± 0.085	17
*Pygospio elegans*	Spiekeroog	80.5 ± 117.397	0.052 ± 0.061	16
*Pygospio elegans*	Wangerooge	16.571 ± 18.155	0.03 ± 0.036	7
*Retusa obtusa*	Norderney	5.786 ± 3.49	0.008 ± 0.009	14
*Retusa obtusa*	Spiekeroog	1.6 ± 0.894	0.006 ± 0.009	5
*Scoloplos armiger*	Norderney	38 ± 54.005	0.245 ± 0.254	15
*Scoloplos armiger*	Spiekeroog	46.615 ± 45.346	0.439 ± 0.494	13
*Scoloplos armiger*	Wangerooge	103.588 ± 93.933	0.124 ± 0.145	17
*Scrobicularia plana*	Norderney	2 ± 0	9.282 ± 0.743	2
*Scrobicularia plana*	Spiekeroog	1 ± NA	5.813 ± NA	1
*Scrobicularia plana*	Wangerooge	1 ± NA	3.297 ± NA	1
*Spio martinensis*	Norderney	1 ± NA	0.001 ± NA	1
*Spionidae* sp.	Spiekeroog	2 ± 1.414	0.001 ± 0.001	2
*Spionidae* sp.	Wangerooge	5.5 ± 2.121	0.01 ± 0.003	2
*Spiophanes bombyx*	Norderney	9 ± NA	0.013 ± NA	1
*Streblospio benedicti*	Norderney	1 ± 0	0.001 ± 0	2
*Streblospio benedicti*	Spiekeroog	20.3 ± 30.405	0.03 ± 0.038	10
*Streblospio benedicti*	Wangerooge	18 ± 2.828	0.028 ± 0.02	2
*Tharyx killariensis*	Norderney	3 ± NA	0.001 ± NA	1
*Tubificoides benedii*	Norderney	130.944 ± 74.937	0.133 ± 0.068	18
*Tubificoides benedii*	Spiekeroog	69.111 ± 72.536	0.133 ± 0.15	18
*Tubificoides benedii*	Wangerooge	83.235 ± 112.279	0.133 ± 0.188	17
*Tubificoides pseudogaster*	Spiekeroog	24 ± 32.527	0.008 ± 0.01	2
*Urothoe poseidonis*	Norderney	4.5 ± 3.697	0.017 ± 0.016	4
*Urothoe poseidonis*	Spiekeroog	10.8 ± 16.884	0.016 ± 0.016	10
*Urothoe poseidonis*	Wangerooge	4.125 ± 3.044	0.012 ± 0.011	8
*Urothoe pulchella*	Wangerooge	6 ± NA	0.001 ± NA	1
*Urothoe* sp.	Spiekeroog	1 ± NA	0 ± NA	1

## References

[ece310815-bib-0001] Anderson, M. J. (2008). Animal‐sediment relationships re‐visited: Characterising species' distributions along an environmental gradient using canonical analysis and quantile regression splines. Journal of Experimental Marine Biology and Ecology, 366(1–2), 16–27. 10.1016/j.jembe.2008.07.006

[ece310815-bib-0002] Armonies, W. (1999). Drifting benthos and long‐term research: Why community monitoring must cover a wide spatial scale. Senckenbergiana Maritima, 29, 13–18.

[ece310815-bib-0003] Armonies, W. (2021). Who lives where? Macrobenthic species distribution over sediment types and depth classes in the eastern North Sea. Helgoland Marine Research, 75(1), 4–9. 10.1186/s10152-021-00552-1

[ece310815-bib-0004] Armonies, W. , & Hellwig‐Armonies, M. (1987). Synoptic patterns of meiofaunal and macrofaunal abundances and specific composition in littoral sediments. Helgoländer Wissenschaftliche Meeresuntersuchungen, 41(1), 83–111.

[ece310815-bib-0005] Bachelet, G. (1990). The choice of a sieving mesh size in the quantitative assessment of marine macrobenthos: A necessary compromise between aims and constraints. Marine Environmental Research, 30(1), 21–35.

[ece310815-bib-0006] Barr, D. J. , Levy, R. , Scheepers, C. , & Tily, H. J. (2013). Random effects structure for confirmatory hypothesis testing: Keep it maximal. Journal of Memory and Language, 68(3), 255–278. 10.1016/j.jml.2012.11.001 PMC3881361 24403724

[ece310815-bib-0007] Bartels‐Hardege, H. D. , & Zeeck, E. (1990). Reproductive behaviour of Nereis diversicolor (Annelida: Polychaeta). Marine Biology, 106, 409–412.

[ece310815-bib-0008] Bates, D. , Mächler, M. , Bolker, B. , & Walker, S. (2015). Fitting linear mixed‐effects models using {lme4}. Journal of Statistical Software, 67(1), 1–48.

[ece310815-bib-0009] Bauer, B. , Berti, E. , Ryser, R. , Gauzens, B. , Hirt, M. R. , Rosenbaum, B. , Digel, C. , Ott, D. , Scheu, S. , & Brose, U. (2022). Biotic filtering by species' interactions constrains food‐web variability across spatial and abiotic gradients. Ecology Letters, 25(5), 1225–1236. 35286010 10.1111/ele.13995

[ece310815-bib-0010] Bearup, D. , & Blasius, B. (2017). Ecotone formation induced by the effects of tidal flooding: A conceptual model of the mud flat‐coastal wetland ecosystem. Ecological Complexity, 32(2016), 217–227. 10.1016/j.ecocom.2016.11.005

[ece310815-bib-0011] Bergfeld, C. (1999). Macrofaunal community pattern in an intertidal Sandflat: Effects of organic enrichment via biodeposition by mussel beds. First results. Senckenbergiana Maritima, 29(Suppl), 23–27.

[ece310815-bib-0012] Bertness, M. D. , & Leonard, G. H. (2014). The role of positive interactions in communities: Lessons from intertidal habitats. Ecology, 78(8), 1976–1989.

[ece310815-bib-0013] Beukema, J. J. (1974). Seasonal changes in the biomass of the macro‐benthos of a tidal flat area in the Dutch Wadden Sea. Netherlands Journal of Sea Research, 8(1), 94–107.

[ece310815-bib-0014] Beukema, J. J. (1976). Biomass and species richness of the macro‐benthic animals living on the tidal flats of the Dutch Wadden Sea. Netherlands Journal of Sea Research, 10(2), 236–261.

[ece310815-bib-0015] Beukema, J. J. , & Cadée, G. C. (1997). Local differences in macrozoobenthic response to enhanced food supply caused by mild eutrophication in a Waddeln Sea area: Food is only locally a limiting factor. Limnology and Oceanography, 42(6), 1424–1435.

[ece310815-bib-0016] Beukema, J. J. , Cadée, G. C. , & Dekker, R. (2002). Zoobenthic biomass limited by phytoplankton abundance: Evidence from parallel changes in two long‐term data series in the Wadden Sea. Journal of Sea Research, 48(2), 111–125.

[ece310815-bib-0017] Beukema, J. J. , & de Vlas, J. (1989). Tidal‐current transport of thread‐drifting postlarval juveniles of the bivalve Macoma balthica from the Wadden Sea to the North Sea. Marine Ecology Progress Series, 52, 193–200.

[ece310815-bib-0018] Beukema, J. J. , & Dekker, R. (2005). Decline of recruitment success in cockles and other bivalves in the Wadden Sea: Possible role of climate change, predation on postlarvae and fisheries. Marine Ecology Progress Series, 287, 149–167.

[ece310815-bib-0019] Beukema, J. J. , & Dekker, R. (2009). The intertidal zoning of cockles (*Cerastoderma edule*) in the Wadden Sea, or why cockle fishery disturbed areas of relatively high biodiversity. Helgoland Marine Research, 63, 287–291.

[ece310815-bib-0020] Beukema, J. J. , & Dekker, R. (2020). Half a century of monitoring macrobenthic animals on tidal flats in the Dutch Wadden Sea. Marine Ecology Progress Series, 656, 1–18. 10.3354/meps13555

[ece310815-bib-0021] Beukema, J. J. , & Dekker, R. (2022). Bottom‐up as well as top‐down processes govern zoobenthic secondary production in a tidal‐flat ecosystem. Limnology and Oceanography, 67(11), 2547–2556.

[ece310815-bib-0022] Beukema, J. J. , Dekker, R. , & Jansen, J. M. (2009). Some like it cold: Populations of the tellinid bivalve *Macoma balthica* (L.) suffer in various ways from a warming climate. Marine Ecology Progress Series, 384, 135–145.

[ece310815-bib-0023] Bosco, J. G. , Thieltges, D. W. , Dekker, R. , Govers, L. L. , Meijer, K. J. , & Klemens Eriksson, B. (2022). Comparing taxonomic and functional trait diversity in marine macrozoobenthos along sediment texture gradients. Ecological Indicators, 145, 109718. 10.1016/j.ecolind.2022.109718

[ece310815-bib-0024] Bouma, H. , Duiker, J. M. C. , De Vries, P. P. , Herman, P. M. J. , & Wolff, W. J. (2001). Spatial pattern of early recruitment of *Macoma balthica* (L.) and *Cerastoderma edule* (L.) in relation to sediment dynamics on a highly dynamic intertidal sandflat. Journal of Sea Research, 45(2), 79–93.

[ece310815-bib-0025] Brooks, M. E. , Kristensen, K. , Van Benthem, K. J. , Magnusson, A. , Berg, C. W. , Nielsen, A. , Skaug, H. J. , Machler, M. , & Bolker, B. M. (2017). glmmTMB balances speed and flexibility among packages for zero‐inflated generalized linear mixed modeling. The R Journal, 9(3), 378–400. 10.32614/RJ-2017-066

[ece310815-bib-0026] Bulling, M. T. , Hicks, N. , Murray, L. , Paterson, D. M. , Raffaelli, D. , White, P. C. L. , & Solan, M. (2010). Marine biodiversity‐ecosystem functions under uncertain environmental futures. Philosophical Transactions of the Royal Society, B: Biological Sciences, 365(1549), 2107–2116. 10.1098/rstb.2010.0022 PMC2880130 20513718

[ece310815-bib-0027] Burson, A. , Stomp, M. , Akil, L. , Brussaard, C. P. D. , & Huisman, J. (2016). Unbalanced reduction of nutrient loads has created an offshore gradient from phosphorus to nitrogen limitation in the North Sea. Limnology and Oceanography, 61(3), 869–888.

[ece310815-bib-0028] Chao, A. , Chiu, C. H. , & Jost, L. (2014). Unifying species diversity, phylogenetic diversity, functional diversity, and related similarity and differentiation measures through hill numbers. Annual Review of Ecology, Evolution, and Systematics, 45, 297–324.

[ece310815-bib-0029] Cheung, W. W. L. , Lam, V. W. Y. , Sarmiento, J. L. , Kearney, K. , Watson, R. , & Pauly, D. (2009). Projecting global marine biodiversity impacts under climate change scenarios. Fish and Fisheries, 10(3), 235–251.

[ece310815-bib-0030] Christianen, M. J. A. , Middelburg, J. J. , Holthuijsen, S. J. , Jouta, J. , Compton, T. J. , van der Heide, T. , Piersma, T. , Sinninghe Damsté, J. S. , van der Veer, H. W. , Schouten, S. , & Olff, H. (2017). Benthic primary producers are key to sustain the Wadden Sea food web: Stable carbon isotope analysis at landscape scale. Ecology, 98(6), 1498–1512. 28369845 10.1002/ecy.1837

[ece310815-bib-0031] Chust, G. , Villarino, E. , Chenuil, A. , Irigoien, X. , Bizsel, N. , Bode, A. , Broms, C. , Claus, S. , Fernández de Puelles, M. L. , Fonda‐Umani, S. , Hoarau, G. , Mazzocchi, M. G. , Mozetič, P. , Vandepitte, L. , Veríssimo, H. , Zervoudaki, S. , & Borja, A. (2016). Dispersal similarly shapes both population genetics and community patterns in the marine realm. Scientific Reports, 6, 28730. 10.1038/srep28730 27344967 PMC4921837

[ece310815-bib-0032] Cloern, J. E. (2001). Our evolving conceptual model of the coastal eutrophication problem. Marine Ecology Progress Series, 210, 223–253.

[ece310815-bib-0033] Colijn, F. , & Dijkema, K. (1981). Species composition of benthic diatoms and distribution of chlorophyll a on an lntertidal flat in the Dutch Wadden Sea. Marine Ecology Progress Series, 4, 9–21.

[ece310815-bib-0034] Compton, T. J. , Holthuijsen, S. , Koolhaas, A. , Dekinga, A. , ten Horn, J. , Smith, J. , Galama, Y. , Brugge, M. , van der Wal, D. , van der Meer, J. , van der Veer, H. W. , & Piersma, T. (2013). Distinctly variable mudscapes: Distribution gradients of intertidal macrofauna across the Dutch Wadden Sea. Journal of Sea Research, 82, 103–116.

[ece310815-bib-0035] Compton, T. J. , Troost, T. A. , Drent, J. , Kraan, C. , Bocher, P. , Leyrer, J. , Dekinga, A. , & Piersma, T. (2009). Repeatable sediment associations of burrowing bivalves across six European tidal flat systems. Marine Ecology Progress Series, 382, 87–98.

[ece310815-bib-0036] Daggers, T. D. , van Oevelen, D. , Herman, P. M. J. , Boschker, H. T. S. , & van der Wal, D. (2020). Spatial variability in macrofaunal diet composition and grazing pressure on microphytobenthos in intertidal areas. Limnology and Oceanography, 65(11), 2819–2834.

[ece310815-bib-0037] de la Vega, C. , Schückel, U. , Horn, S. , Kröncke, I. , Asmus, R. , & Asmus, H. (2018). How to include ecological network analysis results in management? A case study of three tidal basins of the Wadden Sea, South‐Eastern North Sea. Ocean and Coastal Management, 163, 401–416. 10.1016/j.ocecoaman.2018.07.019

[ece310815-bib-0038] Dellwig, O. , Hinrichs, J. , Hild, A. , & Brumsack, H. J. (2000). Changing sedimentation in tidal fiat sediments of the southern north sea from the holocene to the present: A geochemical approach. Journal of Sea Research, 44(3–4), 195–208.

[ece310815-bib-0039] Dewenter, J. , Yong, J. , Schupp, P. J. , Lõhmus, K. , Kröncke, I. , Moorthi, S. , Pieck, D. , Kuczynski, L. , & Rohde, S. (2023). Abundance, biomass and species richness of macrozoobenthos along an intertidal elevation gradient [Dataset]. *Dryad*. 10.5061/dryad.g4f4qrfvd PMC10721958 38107424

[ece310815-bib-0040] Donadi, S. , Eriksson, B. K. , Lettmann, K. A. , Hodapp, D. , Wolff, J. O. , & Hillebrand, H. (2015). The body‐size structure of macrobenthos changes predictably along gradients of hydrodynamic stress and organic enrichment. Marine Biology, 162(3), 675–685.

[ece310815-bib-0041] Dörjes, J. , Michaelis, H. , & Rhode, B. (1986). Long‐term studies of macrozoobenthos in intertidal and shallow subtidal habitats near the island of Norderney (East Frisian coast, Germany). Hydrobiologia, 142(1), 217–232.

[ece310815-bib-0042] Drent, J. (2010). Winter temperature is more important than summer chlorophyll concentrations for macrozoobenthos dynamics in the southern Wadden Sea. Wadden Sea Ecosystem, 26, 97–103.

[ece310815-bib-0043] Dulvy, N. K. , Sadovy, Y. , & Reynolds, J. D. (2003). Extinction vulnerability in marine populations. Fish and Fisheries, 4(1), 25–64.

[ece310815-bib-0044] Eriksson, B. K. , van der Heide, T. , van de Koppel, J. , Piersma, T. , van der Veer, H. W. , & Olff, H. (2010). Major changes in the ecology of the Wadden Sea: Human impacts, ecosystem engineering and sediment dynamics. Ecosystems, 13, 752–764.

[ece310815-bib-0045] Essink, K. , Beukema, J. J. , Madsen, P. B. , Michaelis, H. , & Vedel, G. R. (1998). Long‐term development of biomass of intertidal macrozoobenthos in different parts of the Wadden Sea. Governed by nutrient loads? Senckenbergiana Maritima, 29(1–6), 25–35.

[ece310815-bib-0046] Flemming, B. W. (2000). A revised textural classification of gravel‐free muddy sediments on the basis of ternary diagrams. Continental Shelf Research, 20(10–11), 1125–1137.

[ece310815-bib-0047] Flemming, B. W. (2012). Siliciclastic Back‐barrier tidal flats. In R. A. Davis & R. W. Dalrymple (Eds.), Principles of tidal sedimentology. Springer.

[ece310815-bib-0048] Flemming, B. W. , & Davis, R. A., Jr. (1994). Holocene evolution, morphodynamics and sedimentology of the Spiekeroog barrier Island system (southern North Sea). Senckenbergiana Maritima, 24, 117–155.

[ece310815-bib-0049] Foster, S. D. , & Bravington, M. V. (2013). A Poisson‐gamma model for analysis of ecological non‐negative continuous data. Environmental and Ecological Statistics, 20(4), 533–552.

[ece310815-bib-0050] Fox, J. , Weisberg, S. , Adler, D. , Bates, D. , Baud‐Bovy, G. , Ellison, S. , Firth, D. , Friendly, M. , Gorjanc, G. , Graves, S. , & Heiberger, R. (2012). Package ‘car’. R Foundation for Statistical Computing.

[ece310815-bib-0051] Friedrichs, C. T. (2012). Tidal flat morphodynamics: A synthesis. Treatise on estuarine and coastal science (Vol. 3). Elsevier Inc.

[ece310815-bib-0052] Hagerthey, S. E. , Defew, E. C. , & Paterson, D. M. (2002). Influence of *Corophium volutator* and *Hydrobia ulvae* on intertidal benthic diatom assemblages under different nutrient and temperature regimes. Marine Ecology Progress Series, 245, 47–59.

[ece310815-bib-0053] Hardin, J. W. , & Hilbe, J. M. (2018). Generalized linear models and extensions (4th ed.). Stata Press. https://www.stata‐press.com/books/generalized‐linear‐models‐and‐extensions/

[ece310815-bib-0054] Hartig, F. (2022). DHARMa: Residual Diagnostics for Hierarchical (Multi‐Level /Mixed) Regression Models . R package version 0.4.6. http://florianhartig.github.io/DHARMa

[ece310815-bib-0055] Heip, C. H. R. , Goosen, N. K. , Herman, P. M. J. , Kromkamp, J. , Middelburg, J. J. , & Soetaert, K. (1995). Production and consumption of biological particles in temperate tidal estuaries. Oceanography and Marine Biology: An Annual Review, 33, 1–149.

[ece310815-bib-0056] Hild, A. , Niesel, V. , & Günther, C.‐P. (1999). Study area: The Backbarrier tidal flats of Spiekeroog. In S. Dittmann (Ed.), The Wadden Sea ecosystem (pp. 15–49). Springer.

[ece310815-bib-0057] Hill, N. K. , Woodworth, B. K. , Phinn, S. R. , Murray, N. J. , & Fuller, R. A. (2021). Global protected‐area coverage and human pressure on tidal flats. Conservation Biology, 35(3), 933–943. 32969049 10.1111/cobi.13638 PMC8317051

[ece310815-bib-0058] Hodapp, D. , Kraft, D. , & Hillebrand, H. (2014). Can monitoring data contribute to the biodiversity‐ ecosystem function debate? Evaluating data from a highly dynamic ecosystem. Biodiversity and Conservation, 23, 405–419.

[ece310815-bib-0059] Hoegh‐Guldberg, O. , & Bruno, J. F. (2010). The impact of climate change on the world's marine ecosystems. Science, 328(5985), 1523–1528. 20558709 10.1126/science.1189930

[ece310815-bib-0060] Hope, J. A. , Paterson, D. M. , & Thrush, S. F. (2020). The role of microphytobenthos in soft‐sediment ecological networks and their contribution to the delivery of multiple ecosystem services. Journal of Ecology, 108, 815–830.

[ece310815-bib-0061] Horn, S. , Schwemmer, P. , Mercker, M. , Enners, L. , Asmus, R. , Garthe, S. , & Asmus, H. (2020). Species composition of foraging birds in association with benthic fauna in four intertidal habitats of the Wadden Sea. Estuarine, Coastal and Shelf Science, 233, 106537.

[ece310815-bib-0062] Johnson, P. C. D. (2014). Extension of Nakagawa & Schielzeth's R2GLMM to random slopes models. Methods in Ecology and Evolution, 5(9), 944–946. 25810896 10.1111/2041-210X.12225 PMC4368045

[ece310815-bib-0063] Kark, S. , & van Rensburg, B. J. (2006). Ecotones: Marginal or central areas of transition? Israel Journal of Ecology & Evolution, 52(1), 29–53.

[ece310815-bib-0064] Kondoh, M. (2001). Unifying the relationships of species richness to productivity and disturbance. Proceedings of the Royal Society B: Biological Sciences, 268, 269–271. 10.1098/rspb.2000.1384 PMC1088602 11217897

[ece310815-bib-0065] Kraan, C. , Aarts, G. , van der Meer, J. , & Piersma, T. (2010). The role of environmental variables in structuring landscape‐scale species distributions in seafloor habitats. Ecology, 91(6), 1583–1590. 10.1890/09-2040.1 20583700

[ece310815-bib-0066] Kraft, N. J. B. , Adler, P. B. , Godoy, O. , James, E. C. , Fuller, S. , & Levine, J. M. (2015). Community assembly, coexistence and the environmental filtering metaphor. Functional Ecology, 29(5), 592–599.

[ece310815-bib-0067] Kröncke, I. (1996). Impact of biodeposition on macrofaunal communities in intertidal sandflats. Marine Ecology, 17(1–3), 159–174.

[ece310815-bib-0068] Kröncke, I. (2006). Structure and function of macrofaunal communities influenced by hydrodynamically controlled food availability in the Wadden Sea, the open North Sea, and the deep‐sea. A ssynopsis. Senckenbergiana Maritima, 36(2), 123–164.

[ece310815-bib-0069] Lange, G. , Haynert, K. , Dinter, T. , Scheu, S. , & Kröncke, I. (2018). Adaptation of benthic invertebrates to food sources along marine‐terrestrial boundaries as indicated by carbon and nitrogen stable isotopes. Journal of Sea Research, 131, 12–21.

[ece310815-bib-0070] Le Hir, P. , Roberts, W. , Cazaillet, O. , Christie, M. , Bassoullet, P. , & Bacher, C. (2000). Characterization of intertidal flat hydrodynamics. Continental Shelf Research, 20(12–13), 1433–1459.

[ece310815-bib-0071] Lenhart, H. J. , Mills, D. K. , Baretta‐Bekker, H. , van Leeuwen, S. M. , van der Molen, J. , Baretta, J. W. , Blaas, M. , Desmit, X. , Kühn, W. , Lacroix, G. , Los, H. J. , Ménesguen, A. , Neves, R. , Proctor, R. , Ruardij, P. , Skogen, M. D. , Vanhoutte‐Brunier, A. , Villars, M. T. , & Wakelin, S. L. (2010). Predicting the consequences of nutrient reduction on the eutrophication status of the North Sea. Journal of Marine Systems, 81, 148–170.

[ece310815-bib-0072] Li, D. (2018). hillR: Taxonomic, functional, and phylogenetic diversity and similarity through Hill numbers. Journal of Open Source Software, 3(31), 1041.

[ece310815-bib-0073] Linton, D. L. , & Taghon, G. L. (2000). Feeding, growth, and fecundity of *Capitella* sp. I in relation to sediment organic concentration. Marine Ecology Progress Series, 205, 229–240. 10.1016/s0022-0981(00)00266-5 11058728

[ece310815-bib-0074] Lotze, H. K. (2005). Radical changes in the Wadden Sea fauna and flora over the last 2,000 years. Helgoland Marine Research, 59(1), 71–83.

[ece310815-bib-0075] Lotze, H. K. , Lenihan, H. S. , Bourque, B. J. , Bradbury, R. H. , Cooke, R. G. , Kay, M. C. , Kidwell, S. M. , Kirby, M. X. , Peterson, C. H. , & Jackson, J. B. C. (2006). Depletion, degradation and recovery potential of Esutaries and coastal seas. Science, 312(5781), 1806–1809. 16794081 10.1126/science.1128035

[ece310815-bib-0076] Lüdecke, D. (2022). ‘sjPlot: Data Visualization for Statistics in Social Science . R package version 2.8.12. https://CRAN.‐Rproject.org/package=sjPlot

[ece310815-bib-0077] MacIntyre, H. L. , Geider, R. J. , & Miller, D. C. (1996). Microphytobenthos: The ecological role of the “secret garden” of Unvegetated, shallow‐water marine habitats. I. Distribution, abundance and primary production. Estuaries, 19(2A), 186–201.

[ece310815-bib-0079] Meier, D. , Thölen, C. , Hillebrand, H. , Kleyer, M. , Lõhmus, K. , & Zielinski, O. (2020). Continuous temperature observations in surface sediment within DynaCom experimental islands and saltmarsh enclosed plots at different elevation levels, Spiekeroog, Germany, 2019‐01 to 2019‐12. PANGAEA.

[ece310815-bib-0080] Meyerjürgens, J. , Badewien, T. H. , Garaba, S. P. , Wolff, J. O. , & Zielinski, O. (2019). A state‐of‐the‐art compact surface drifter reveals pathways of floating marine litter in the German bight. Frontiers in Marine Science, 6, 1–15.36817748

[ece310815-bib-0081] Meysman, F. J. R. , Galaktionov, O. S. , Gribsholt, B. , & Middelburg, J. J. (2006). Bioirrigation in permeable sediments: Advective pore‐water transport induced by burrow ventilation. Limnology and Oceanography, 51(1 I), 142–156.

[ece310815-bib-0082] Mouquet, N. , & Loreau, M. (2003). Community patterns in source‐sink Metacommunities. American Naturalist, 162(5), 544–557. 10.1086/378857 14618534

[ece310815-bib-0083] Murray, N. J. , Phinn, S. R. , DeWitt, M. , Ferrari, R. , Johnston, R. , Lyons, M. B. , Clinton, N. , Thau, D. , & Fuller, R. A. (2019). The global distribution and trajectory of tidal flats. Nature, 565(7738), 222–225. 30568300 10.1038/s41586-018-0805-8

[ece310815-bib-0084] Nakagawa, S. , Johnson, P. C. D. , & Schielzeth, H. (2017). The coefficient of determination *R* ^2^ and intra‐class correlation coefficient from generalized linear mixed‐effects models revisited and expanded. Journal of the Royal Society Interface, 14(134), 20170213. 28904005 10.1098/rsif.2017.0213 PMC5636267

[ece310815-bib-0085] Nehmer, P. , & Kröncke, I. (2003). Macrofaunal communities in the Wichter Ee, a channel system in the East Frisian Wadden Sea. Senckenbergiana Maritima, 32(1/2), 1–10.

[ece310815-bib-0086] Niku, J. , Warton, D. I. , Hui, F. K. C. , & Taskinen, S. (2017). Generalized linear latent variable models for multivariate count and biomass data in ecology. Journal of Agricultural, Biological, and Environmental Statistics, 22(4), 498–522.

[ece310815-bib-0087] NLWKN . (2010). Generalplan Küstenschutz Niedersachsen – Ostfriesische Inseln. Norden.

[ece310815-bib-0088] Oksanen, J. , Simpson, G. L. , Blanchet, F. G. , Kindt, R. , Legendre, P. , Minchin, P. R. , O'Hara, R. B. , Solymos, P. , Stevens, M. H. H. , Szoecs, E. , Wagner, H. , Barbour, M. , Bedward, M. , Bolker, B. , Borcard, D. , Carvalho, G. , Chirico, M. , De Caceres, M. , Durand, S. , … Weedon, J. (2007). The vegan package. Community Ecology Package, 10(631–637), 719.

[ece310815-bib-0089] Otto, L. , Zimmerman, J. T. F. , Furnes, G. K. , Mork, M. , Saetre, R. , & Becker, G. (1990). Review of the physical oceanography of the North Sea. Netherlands Journal of Sea Research, 26(2–4), 161–238.

[ece310815-bib-0090] Palmer, M. A. , Allan, J. D. , & Butman, C. A. (1996). Dispersal as a regional process affecting the local dynamics of marine and stream benthic invertebrates. Trends in Ecology & Evolution, 11, 322–326. 21237862 10.1016/0169-5347(96)10038-0

[ece310815-bib-0091] Pearson, T. H. , & Rosenberg, R. (1978). Macrobenthic succession in relation to organic enrichment and pollution of the marine environment. Oceanography and Marine Biology: An Annual Review, 16, 229–311.

[ece310815-bib-0092] Philippart, C. J. M. , Beukema, J. J. , Cadée, G. C. , Dekker, R. , Goedhart, P. W. , van Iperen, J. M. , Leopold, M. F. , & Herman, P. M. J. (2007). Impacts of nutrient reduction on coastal communities. Ecosystems, 10, 95–118.

[ece310815-bib-0093] Pratt, D. R. , Pilditch, C. A. , Lohrer, A. M. , Thrush, S. F. , & Kraan, C. (2015). Spatial distributions of grazing activity and microphytobenthos reveal scale‐dependent relationships across a sedimentary gradient. Estuaries and Coasts, 38(3), 722–734.

[ece310815-bib-0094] Puls, W. , van Bernem, K.‐H. , Eppel, D. , Kapitza, H. , Pleskachevsky, A. , Riethmüller, R. , & Vaessen, B. (2012). Prediction of benthic community structure from environmental variables in a soft‐sediment tidal basin (North Sea). Helgoland Marine Research, 66, 345–361.

[ece310815-bib-0095] R Core Team . (2021). R: A language and environment for statistical computing. R Foundation for Statistical Computing. https://www.r‐project.org/

[ece310815-bib-0096] Reineck, H. E. , & Siefert, W. (1980). Faktoren der Schlickbildung im Sahlenburger und Neuwerker Watt. Die Küste, 35, 26–51.

[ece310815-bib-0097] Reise, K. , Herre, E. , & Sturm, M. (1994). Biomass and abundance of macrofauna in intertidal sediments of Königshafen in the northern Wadden Sea. Helgoländer Wissenschaftliche Meeresuntersuchungen, 48(2–3), 201–215.

[ece310815-bib-0098] Reise, K. (2002). Sediment mediated species interactions in coastal waters. Journal of Sea Research, 48(2), 127–141.

[ece310815-bib-0099] Reiss, H. , & Kröncke, I. (2001). Spatial and temporal distribution of macrofauna in the Otzumer Balje (east Frisian Wadden Sea, Germany). Senckenbergiana Maritima, 31(2), 283–298.

[ece310815-bib-0100] Respondek, G. , Günther, C. , Beier, U. , Bleeker, K. , Pedersen, E. M. , Schulze, T. , & Temming, A. (2022). Connectivity of local sub‐stocks of *Crangon crangon* in the North Sea and the risk of local recruitment overfishing. Journal of Sea Research, 181, 102173. 10.1016/j.seares.2022.102173

[ece310815-bib-0101] Riekenberg, P. , van der Heide, T. , Holthuijsen, S. J. , van der Veer, H. W. , & van der Meer, M. T. J. (2022). Compound‐specific stable isotope analysis of amino acid nitrogen reveals detrital support of microphytobenthos in the Dutch Wadden Sea benthic food web. Frontiers in Ecology and Evolution, 10, 1–29.

[ece310815-bib-0132] RStudio Team . (2022). RStudio: Integrated Development Environment for R . RStudio, PBC, Boston, MA. http://www.rstudio.com/

[ece310815-bib-0102] Schückel, U. , Beck, M. , & Kröncke, I. (2013). Spatial variability in structural and functional aspects of macrofauna communities and their environmental parameters in the Jade Bay (Wadden Sea Lower Saxony, southern North Sea). Helgoland Marine Research, 67(1), 121–136.

[ece310815-bib-0103] Schückel, U. , & Kröncke, I. (2013). Temporal changes in intertidal macrofauna communities over eight decades: A result of eutrophication and climate change. Estuarine, Coastal and Shelf Science, 117, 210–218.

[ece310815-bib-0104] Schückel, U. , Kröncke, I. , & Baird, D. (2015). Linking long‐term changes in trophic structure and function of an intertidal macrobenthic system to eutrophication and climate change using ecological network analysis. Marine Ecology Progress Series, 536, 25–38.

[ece310815-bib-0105] Sijtsma, F. J. , Mehnen, N. , Angelstam, P. , & Muñoz‐Rojas, J. (2019). Multi‐scale mapping of cultural ecosystem services in a socio‐ecological landscape: A case study of the international Wadden Sea region. Landscape Ecology, 34(7), 1751–1768.

[ece310815-bib-0106] Singer, A. , Bijleveld, A. I. , Hahner, F. , Holthuijsen, S. J. , Hubert, K. , Kerimoglu, O. , Kleine Schaars, L. , Kröncke, I. , Lettmann, K. A. , Rittweg, T. , Scheiffarth, G. , van der Veer, H. W. , & Wurpts, A. (2023). Long‐term response of coastal macrofauna communities to de‐ eutrophication and sea level rise mediated habitat changes (1980s versus 2018). Frontiers in Marine Science, 9, 1–20.

[ece310815-bib-0107] Sprung, M. , Asmus, H. , & Asmus, R. (2001). Energy flow in benthic assemblages of tidal basins: Ria Formosa (Portugal) and Sylt‐Rømø Bay (North Sea) compared. In K. Reise (Ed.), Ecological comparisons of sedimentary shores (pp. 237–254). Springer.

[ece310815-bib-0108] Stanev, E. V. , Brink‐Spalink, G. , & Wolff, J. O. (2007). Sediment dynamics in tidally dominated environments controlled by transport and turbulence: A case study for the east Frisian Wadden Sea. Journal of Geophysical Research: Oceans, 112(4), 1–20.

[ece310815-bib-0109] Stanev, E. V. , Wölff, J. O. , Burchard, H. , Bolding, K. , & Flöser, G. (2003). On the circulation in the east Frisian Wadden Sea: Numerical modeling and data analysis. Ocean Dynamics, 53(1), 27–51.

[ece310815-bib-0110] Staneva, J. , Stanev, E. V. , Wolff, J. O. , Badewien, T. H. , Reuter, R. , Flemming, B. , Bartholomä, A. , & Bolding, K. (2009). Hydroynamics and sediment dynamics in the German bight. A focus on observations and numerical modelling in the east Frisian Wadden Sea. Continental Shelf Research, 29(1), 302–319.

[ece310815-bib-0111] Sutherland, W. J. , Freckleton, R. P. , Godfray, H. C. J. , Beissinger, S. R. , Benton, T. , Cameron, D. D. , Carmel, Y. , Coomes, D. A. , Coulson, T. , Emmerson, M. C. , Hails, R. S. , Hays, G. C. , Hodgson, D. J. , Hutchings, M. J. , Johnson, D. , Jones, J. P. G. , Keeling, M. J. , Kokko, H. , Kunin, W. E. , … Wiegand, T. (2013). Identification of 100 fundamental ecological questions. Journal of Ecology, 101(1), 58–67.

[ece310815-bib-0112] Thrane, J. E. , Kyle, M. , Striebel, M. , Haande, S. , Grung, M. , Rohrlack, T. , & Andersen, T. (2015). Spectrophotometric analysis of pigments: A critical assessment of a high‐throughput method for analysis of algal pigment mixtures by spectral deconvolution. PLoS One, 10(9), e0137645. 26359659 10.1371/journal.pone.0137645 PMC4567325

[ece310815-bib-0113] Underwood, G. J. C. , & Kromkamp, J. (1999). Primary production by phytoplankton and microphytobenthos in estuaries. Advances in Ecological Research, 29, 93–154.

[ece310815-bib-0114] van Beusekom, J. E. E. , Bot, P. V. M. , Carstensen, J. , Goebel, J. H. , Lenhart, H. , Pätsch, J. , Petenati, T. , Raabe, T. , Reise, K. , & Wetsteijn, B. (2009). Eutrophication. Thematic report No. 6. In H. Marencic & J. de Vlas (Eds.), Quality status report 2009. Wadden Sea ecosystem No. 25. Wilhelmshaven, Germany.

[ece310815-bib-0115] van Beusekom, J. E. E. , Carstensen, J. , Dolch, T. , Grage, A. , Hofmeister, R. , Lenhart, H. , Kerimoglu, O. , Kolbe, K. , Pätsch, J. , Rick, J. , Rönn, L. , & Ruiter, H. (2019). Wadden Sea eutrophication: Long‐term trends and regional differences. Frontiers in Marine Science, 6(370), 1–17.36817748

[ece310815-bib-0116] Van Colen, C. , Vincx, M. , Herman, P. M. J. , Ysebaert, T. , & Degraer, S. (2010). Macrobenthos recruitment success in a tidal flat: Feeding trait dependent effects of disturbance history. Journal of Experimental Marine Biology and Ecology, 385(1–2), 79–84.

[ece310815-bib-0117] Van Colen, C. , Rossi, F. , Montserrat, F. , Andersson, M. G. I. , Gribsholt, B. , Herman, P. M. J. , Degraer, S. , Vincx, M. , Ysebaert, T. , & Middelburg, J. J. (2012). Organism‐sediment interactions govern post‐hypoxia recovery of ecosystem functioning. PLoS One, 7(11), e49795. 23185440 10.1371/journal.pone.0049795 PMC3504103

[ece310815-bib-0118] van der Wal, D. , Lambert, G. I. , Ysebaert, T. , Plancke, Y. M. G. , & Herman, P. M. J. (2017). Hydrodynamic conditioning of diversity and functional traits in subtidal estuarine macrozoobenthic communities. Estuarine, Coastal and Shelf Science, 197, 80–92. 10.1016/j.ecss.2017.08.012

[ece310815-bib-0119] van der Wal, D. , Ysebaert, T. , & Herman, P. M. J. (2017). Response of intertidal benthic macrofauna to migrating megaripples and hydrodynamics. Marine Ecology Progress Series, 585, 17–30.

[ece310815-bib-0121] van Raaphorst, W. , & de Jonge, V. N. (2004). Reconstruction of the total N and P inputs from the IJsselmeer into the western Wadden Sea between 1935–1998. Journal of Sea Research, 51, 109–131.

[ece310815-bib-0122] van Roomen, M. , Laursen, K. , van Turnhout, C. , van Winden, E. , Blew, J. , Eskildsen, K. , Günther, K. , Hälterlein, B. , Kleefstra, R. , Potel, P. , Schrader, S. , Luerssen, G. , & Ens, B. J. (2012). Signals from the Wadden Sea: Population declines dominate among waterbirds depending on intertidal mudflats. Ocean and Coastal Management, 68, 79–88. 10.1016/j.ocecoaman.2012.04.004

[ece310815-bib-0123] Weerman, E. J. , Herman, P. M. J. , & Van De Koppel, J. (2011). Top‐down control inhibits spatial self‐organization of a patterned landscape. Ecology, 92(2), 487–495. 21618927 10.1890/10-0270.1

[ece310815-bib-0124] Wickham, H. , Chang, W. , & Wickham, M. H. (2016). Package ‘ggplot2.’ *Create elegant data visualisations using the grammar of graphics* . Version, 2(1), 1–189.

[ece310815-bib-0125] Willis, K. J. , & Whittaker, R. J. (2002). Species diversity – Scale matters. Science, 295(5558), 1245–1248. 11847328 10.1126/science.1067335

[ece310815-bib-0126] Worm, B. , Barbier, E. B. , Beaumont, N. , Duffy, J. E. , Folke, C. , Halpern, B. S. , Jackson, J. B. C. , Lotze, H. K. , Micheli, F. , Palumbi, S. R. , Sala, E. , Selkoe, K. A. , Stachowicz, J. J. , & Watson, R. (2006). Impacts of biodiversity loss on ocean ecosystem services. Science, 316(5829), 787–791. 10.1126/science.1132294 17082450

[ece310815-bib-0127] Yong, J. , Moick, M. , Dewenter, J. , Hillebrand, H. , Kröncke, I. , Rohde, S. , & Moorthi, S. (2022). Spatial and temporal patterns of microphytobenthos communities along the marine‐terrestrial boundary in the German Wadden Sea. Frontiers in Ecology and Evolution, 10, 1–21.

[ece310815-bib-0128] Ysebaert, T. , Herman, P. M. J. , Meire, P. , Craeymeersch, J. , Verbeek, H. , & Heip, C. H. R. (2003). Large‐scale spatial patterns in estuaries: Estuarine macrobenthic communities in the Schelde estuary, NW Europe. Estuarine, Coastal and Shelf Science, 57(1–2), 335–355.

[ece310815-bib-0129] Ysebaert, T. , & Herman, P. M. J. (2002). Spatial and temporal variation in benthic macrofauna and relationships with environmental variables in an estuarine, intertidal soft‐sediment environment. Marine Ecology Progress Series, 244, 105–124.

[ece310815-bib-0130] Zielinski, O. , Meier, D. , Kleyer, M. , Lõhmus, K. , & Hillebrand, H. (2019). Continuous meteorological observations at DynaCom automatic weather station (2019), Spiekeroog, Germany, 2019‐01 to 2019‐09. *PANGAEA* .

[ece310815-bib-0131] Zuur, A. F. , Ieno, E. N. , & Elphick, C. S. (2010). A protocol for data exploration to avoid common statistical problems. Methods in Ecology and Evolution, 1(1), 3–14.

